# Principal component analysis-based unsupervised feature extraction applied to *in silico* drug discovery for posttraumatic stress disorder-mediated heart disease

**DOI:** 10.1186/s12859-015-0574-4

**Published:** 2015-04-30

**Authors:** Y-h Taguchi, Mitsuo Iwadate, Hideaki Umeyama

**Affiliations:** 1 Department of Physics, Chuo University, 1-13-27 Kasuga, Bunkyo-ku, Tokyo, 112-8551 Japan; 2 Department of Biological Science, Chuo University, 1-13-27 Kasuga, Bunkyo-ku, Tokyo, 112-8551 Japan

**Keywords:** Unsupervised feature extraction, Principal component analysis, Variational Bayes, Posttraumatic stress disorder, Heart disease, *In silico* drug discovery, chooseLD, FAMS

## Abstract

**Background:**

Feature extraction (FE) is difficult, particularly if there are more features than samples, as small sample numbers often result in biased outcomes or overfitting. Furthermore, multiple sample classes often complicate FE because evaluating performance, which is usual in supervised FE, is generally harder than the two-class problem. Developing sample classification independent unsupervised methods would solve many of these problems.

**Results:**

Two principal component analysis (PCA)-based FE, specifically, variational Bayes PCA (VBPCA) was extended to perform unsupervised FE, and together with conventional PCA (CPCA)-based unsupervised FE, were tested as sample classification independent unsupervised FE methods. VBPCA- and CPCA-based unsupervised FE both performed well when applied to simulated data, and a posttraumatic stress disorder (PTSD)-mediated heart disease data set that had multiple categorical class observations in mRNA/microRNA expression of stressed mouse heart. A critical set of PTSD miRNAs/mRNAs were identified that show aberrant expression between treatment and control samples, and significant, negative correlation with one another. Moreover, greater stability and biological feasibility than conventional supervised FE was also demonstrated. Based on the results obtained, *in silico* drug discovery was performed as translational validation of the methods.

**Conclusions:**

Our two proposed unsupervised FE methods (CPCA- and VBPCA-based) worked well on simulated data, and outperformed two conventional supervised FE methods on a real data set. Thus, these two methods have suggested equivalence for FE on categorical multiclass data sets, with potential translational utility for *in silico* drug discovery.

**Electronic supplementary material:**

The online version of this article (doi:10.1186/s12859-015-0574-4) contains supplementary material, which is available to authorized users.

## Background

Feature extraction (FE) is an important task in bioinformatic analyses, as there are often more features than samples. The number of bases spanning linear space is at most equivalent to the number of independent vectors. Accordingly, more features than samples inevitably leads to redundancy. Although dimensional reduction is often used to eliminate redundancy, it is far from true redundancy elimination as reconstructed bases are usually the linear combination of all features, which is not always necessary for spanning entire linear space.

Instead of dimensional reduction, FE can be used to eliminate redundancy, and is often performed to maximize performance of targeted tasks (supervised FE), e.g., discrimination between samples or regression analysis, although fewer samples than features often creates difficulties due to overfitting and/or bias. Multiple class samples commonly provide additional problems when supervised FE is used, complicating performance evaluations compared with two-class samples. Although pairwise evaluations (e.g., one versus one or one versus others) are possible, they are time-consuming. FE using a set of pairwise evaluations is often even more difficult, because it is hard to maximize performance simultaneously for all pairwise evaluations with commonly selected features for all pairs. In addition, supervised FE is often heavily sample-dependent, and alternative sample sets often provide alternative optimal FE. Moreover, with categorical classes the problems are greater, since frequently used FE with regression analyses, e.g., lasso [1], cannot be directly applied to categorical multiclass data sets.

In order to avoid these difficulties, unsupervised FE is useful as it is assumed to be more robust and stable, although this has not been extensively studied because of implementation difficulties, e.g., supervision is not based upon performance, therefore FE has nothing suitable for optimization. Variational Bayesian approaches eliminate redundancy in an unsupervised manner, and have recently been proposed as promising unsupervised FE methods. In this paper, we used variational Bayes (VB) principal component analysis (PCA) [2,3] to perform unsupervised FE. In addition, a simpler and more conventional PCA (CPCA)-based unsupervised FE method (CPCAFE) that worked well with a simulated data set was proposed as an alternative to VBPCA. As VBPCA is more computationally challenging, CPCAFE is an even more promising alternative unsupervised FE candidate than the VB approach.

To demonstrate applicability to translational research, we used CPCA-based, and partially, VBPCA-based unsupervised FE (VBPCAFE) to examine heart disease associated with posttraumatic stress disorder (PTSD). PTSD [4] is caused by exposure to high-magnitude, life-threatening stressors, i.e., traumatic events. PTSD patients typically develop acute stress responses that include symptoms of arousal, anxiety, sadness, grief, agitation, irritability, and sleep disturbances. Heart disease is associated with PTSD, with a meta-analysis showing association between coronary heart disease (CHD) and PTSD [5]. Moreover, hospitalization due to cardiovascular disease is associated with PTSD caused by the September 11, 2001, World Trade Center disaster [6]. PTSD was also associated with CHD using a prospective twin study design [7]. However, the underlying genetic background of the association is not well known.

By applying CPCAFE and VBPCAFE to publically available mRNA and microRNA (miRNA) expression data [8] from stressed mouse hearts, we identified aberrantly expressed miRNAs and mRNAs. Biological feasibility of the identified miRNAs and mRNAs was determined (by negative correlation between miRNAs and mRNAs, and Kyoto Encyclopedia of Genes and Genomes (KEGG) [9] pathway enrichment of miRNA target genes), showing suitability of our selections.

Two supervised FE methods, specifically, regression analysis using categorical pseudo variables and backward elimination using Hilbert-Schmidt norm of the cross-covariance operator (BAHSIC), identified unstable and biologically less feasible sets of mRNAs and miRNAs that were not always negatively correlated, and suggested superiority of unsupervised FE methods.

Among the aberrantly expressed genes identified by CPCAFE, fatty acid binding protein 3 (FABP3) was considered a potential drug target candidate by structural investigations, and inhibitory drugs were sought using the *in silico* drug discovery tool, chooseLD, a profile-based drug discovery program.

## Results and discussion

### FE methods applied to a simulated categorical multiclass data set

We performed FE using a simulated categorical multiclass data set, to determine the limitations of FE and usefulness of our proposed methods.

**Figure 1:**
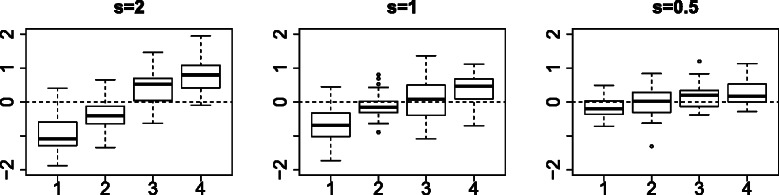
Boxplot of typical features with distinct values between four classes (1≤*i*≤5) in the simulated data set; *s*=2 (easy), 1 (medium), and 0.5 (hard).

The data set consisted of 100 simulated ensembles of 20 samples with 100 features, of which only 10 features were distinct between four classes, and with each class consisting of 5 samples (see Methods). As sample values of each feature were obtained from an identical mixture of four Gaussian distributions, FE is difficult but discrimination between four classes varies between three cases (shown in Figure 1; a typical feature boxplot with distinct expression among classes). We named these three cases as easy (*s*=2), medium (*s*=1), and hard (*s*=0.5). Furthermore, we did not use order between the four classes, so the data could be treated as a categorical data set.

**Figure 2:**
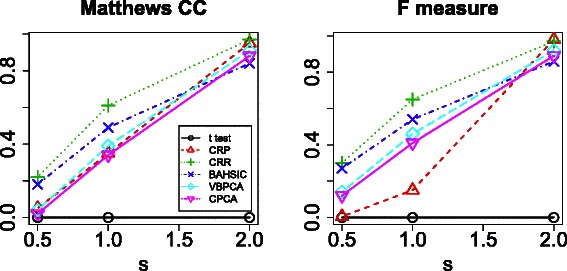
Matthews correlation coefficients and *F* measure for various FE methods applied to the simulated data set.*t* test, one vs one *t* test based FE; CRP, categorical regression based FE (using adjusted *P*-values); CRR, categorical regression based FE (using ranked *P*-values); BAHSIC, backward elimination using Hilbert-Schmidt norm of the cross-covariance operator; VBPCAFE, variational Bayes principal component analysis based unsupervised FE; CPCAFE, conventional principal component analysis based unsupervised FE.

In order to demonstrate the limitations of FE, we first tested one vs one *t* test based FE (see Methods). No significantly different features were detected between the four classes in any of the three cases (Figure 2; confusion tables are available in Additional file 1), demonstrating that FE on a categorical multiclass data set is difficult.

Next, we applied a more sophisticated method, specifically, categorical regression based FE (using significance based on adjusted *P*-values, see Methods). The number of correctly extracted features improved, with Matthews correlation coefficients of 0.95, 0.35, and 0.05, and *F* measures of 0.98, 0.15, and 0.005 for easy, medium, and hard cases, respectively (Figure 2; confusion tables are available in Additional file 1). However, for the hard case, both values are almost zero.

In order to improve performance, we selected the top 10 ranked features with the smallest *P*-values, independent of significance (using *P*-value ranks, see Methods). This resulted in Matthews correlation coefficients of 0.97, 0.61, and 0.22, and *F* measures of 0.97, 0.65, and 0.30 for easy, medium, and hard cases, respectively (Figure 2; confusion tables are available in Additional file 1). Thus, FE accuracy is reasonable, but selecting features using non-significant *P*-values is not ideal, highlighting the problem of using FE on a categorical multiclass data set.

In order to overcome this, we next tested BAHSIC [10] (see Methods), a recently proposed non-parametric FE method that has been used on high-dimensional microarray data sets. The obtained Matthews correlation coefficients were 0.84, 0.49, and 0.18, and *F* measures were 0.86, 0.54, and 0.27 for easy, medium, and hard cases, respectively (Figure 2; confusion tables are available in Additional file 1). Although performance is relatively improved (without using highly ranked but non-significant features), BAHSIC does not use *P*-values, and again the results are not completely satisfactory, especially for the easy case: a Matthews coefficient of 0.84 is less than the 0.95 achieved by categorical regression based FE.

**Figure 3:**
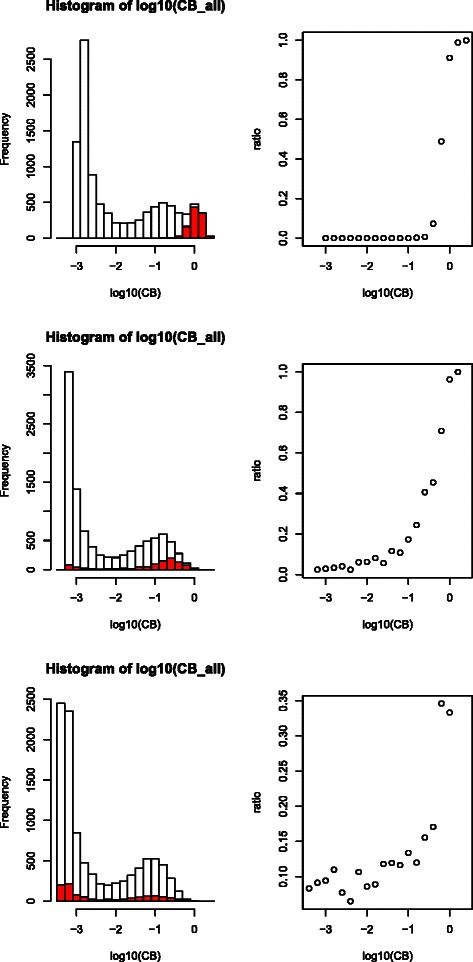
Relationship between ${C_{b}^{i}}$ and features with distinct expression among four classes. Left column: histogram obtained from logarithmic ${C_{b}^{i}}$ for 100 independent ensembles. Red color indicates features with distinct expression between four classes. Right column: proportion of features with distinct expression between four classes in each bin. Top: *s*=2, easy; middle: *s*=1, medium; bottom: *s*=0.5, hard cases.

**Figure 4:**
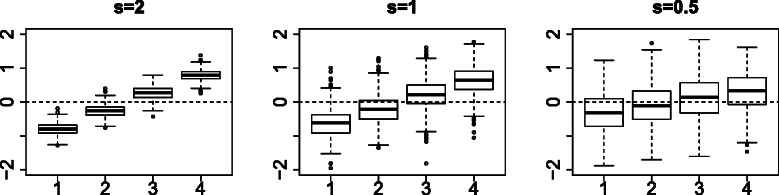
*A*_*j*1_ boxplot with distinct values between four classes in 100 independent ensembles of the simulated data set; *s*=2 (easy), 1 (medium), and 0.5 (hard).

Therefore, we next considered VBPCAFE. In order to perform FE with VBPCA, original VBPCA was extended to incorporate feature dependence (see Methods). Prior distribution in this extension has feature-dependent parameters, and therefore automatic elimination of irrelevant features was expected. The $C_{B}^{i1}$ histogram reflects the prior distribution parameter of the first principal component (PC) (Figure 3), with smaller values assumed to be irrelevant features. Originally, *C*_*B*_ was introduced to eliminate irrelevant PCs, and therefore has only *q* (PC) and not *i* (feature) dependence. However, we needed to eliminate irrelevant features as well; therefore, in our study *C*_*B*_ must also have *i* dependence. Although we did not use sample labelling information, as expected, irrelevant features had smaller $C_{B}^{i1}$. Averaged *A*_*j*1_ (contribution of the *j*th sample to the first PC) over 100 ensembles (Figure 4), demonstrates that *A*_*j*1_ represents the distinction between the four classes. Thus, features not coincident with *A*_*j*1_ (and with smaller $C_{B}^{i1}$) were eliminated, producing Matthews correlation coefficients of 0.91, 0.39, and 0.04, and *F* measures of 0.92, 0.46, and 0.14 for easy, medium, and hard cases, respectively (Figure 2; confusion tables are available in Additional file 1).

Although performance has been successfully improved for the easy case, VBPCAFE is computationally challenging and is a potential drawback to its use. Iteration involves updates proportional to the number of features, which can often be as many as several tens of thousands. Thus, a more computationally effective method is of value, and a relatively straightforward idea is to replace $C_{B}^{iq}$ with *B*_*iq*_, the *q*th PC score of *i*th feature, because a larger *B*_*iq*_ is expected to result in relevant (thus larger) $C_{B}^{iq}$ (for example, see eq. (1) in Methods). Indeed, replacing $C_{B}^{i1}$ with *B*_1*i*_, we obtained Matthews correlation coefficients of 0.88, 0.34, and 0.02, and *F* measures of 0.89, 0.41, and 0.12 for easy, medium and hard cases, respectively (Figure 2, confusion tables are available in Additional file 1). These values are comparable with those using $C_{B}^{i1}$. Considering that CPCAFE requires minimal computational resources, CPCAFE is a promising candidate for applying to real data that often consists of more than several thousand features.

In summary, there are at least four methods that achieve comparable FE performance on a categorical multiclass data set: categorical regression based FE (using ranked *P*-values), BAHSIC, VBPCAFE, and CPCAFE. Each has its own advantages and disadvantages: although categorical regression based FE achieved the best overall performance, it used features with non-significant adjusted *P*-values. BAHSIC does not have this problem, but as it does not use *P*-values, its performance using the easy case was poorest of the tested methods. VBPCAFE and CPCAFE were more effective using the easy case, but their performance was poor for the hard case. VBPCAFE is computationally challenging, while CPCAFE is not.

However, these conclusions may be viewed as slightly subjective. The methods recommended for the hard case (i.e., categorical regression based FE and BAHSIC) have strict label dependency. Harder class discrimination often means label uncertainty (or incorrectness). Of course, there are many objective labels (e.g., gender or age), but those related to experimental conditions often include subjective criterion or insufficient controls for experimental conditions. Hard class discrimination often originates from these subjective labels, and consequently, robustness to mislabeling is desired, especially for hard cases.

**Figure 5:**
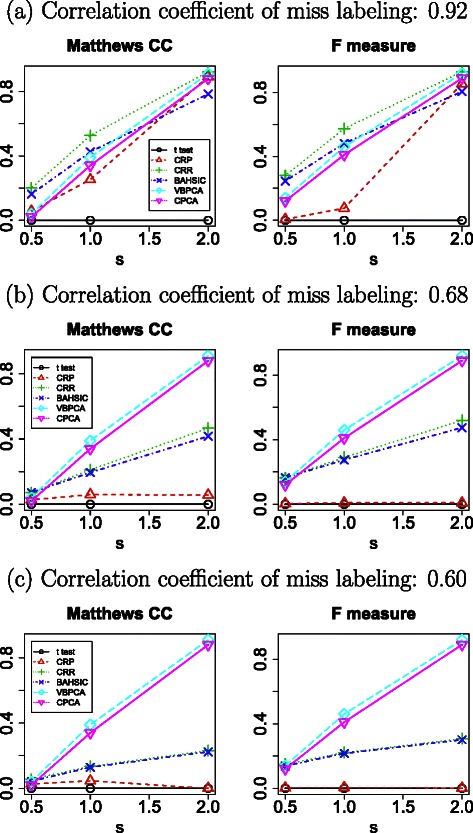
Matthews correlation coefficients and *F* measure for various FE methods applied to the simulated data set with partial mislabeling, indicated in Table 1. Correlation coefficients between mislabeled and true labeling were **(a)** 0.92, **(b)** 0.68, and **(c)** 0.60, as shown in Table 1. Other notations are the same as in Figure 2.

In order to test robustness of the four methods towards partial mislabeling, we determined performance after mislabeling (Table 1). Figure 5 shows performance degradation caused by little, medium, and heavy partial mislabeling. Little, medium, and heavy were defined by the distance between true and wrong labels. The number of samples with wrong labels included only a proportion of the data, and never more than 20%. Although only 20% of samples were mislabeled, performance degradation with BAHSIC and categorical regression based FE was considerable for medium and heavy mislabeling. Conversely, VBPCAFE and CPNAFE performance were not affected by mislabeling as these two methods do not require labeling information. This indicates that the two methods recommended for hard cases are not particularly robust, even against partial mislabeling, and therefore not always recommended.

**Table 1:** **Artificial partial mislabeling introduced to the simulated data set to check robustness of FE**

	**(a)**					**(b)**					**(c)**			
**Corr. coef.**	**0.92**					**0.68**					**0.60**			
**Class**	**1**	**2**	**3**	**4**		**1**	**2**	**3**	**4**		**1**	**2**	**3**	**4**
1	4	1	0	0		4	0	1	0		4	0	0	1
2	1	4	0	0		0	4	0	1		0	4	1	0
3	0	0	4	1		1	0	4	0		0	1	4	0
4	0	0	1	4		0	1	0	4		1	0	0	4

Rows and columns represent true and modified labels. Numbers represent the number of samples with correct and modified labels. (a) little; (b) medium; and (c) heavy mislabeling. Correlation coefficients represent the amount of mislabeling (larger correlation coefficients correspond to less mislabeling).

In conclusion, CPCAFE or VBPCAFE are the best methods for FE from categorical multiclass data sets, as they maintain robustness and show relatively stable and good performance for most cases.

### Translational use of CPCAFE

**Figure 6:**
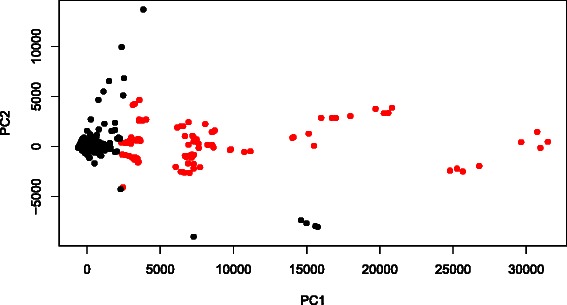
Two-dimensional embedding of miRNA expression determined by PCA. Each dot represents a probe. Red dots indicate the 100 top-ranked probes with larger PC1 scores, selected as outliers based on the criterion of CPCAFE. A few probes with relatively large PC2 scores were excluded to allow selection of PC1 enhanced probes. See Additional file 2 for more detail.

**Figure 7:**
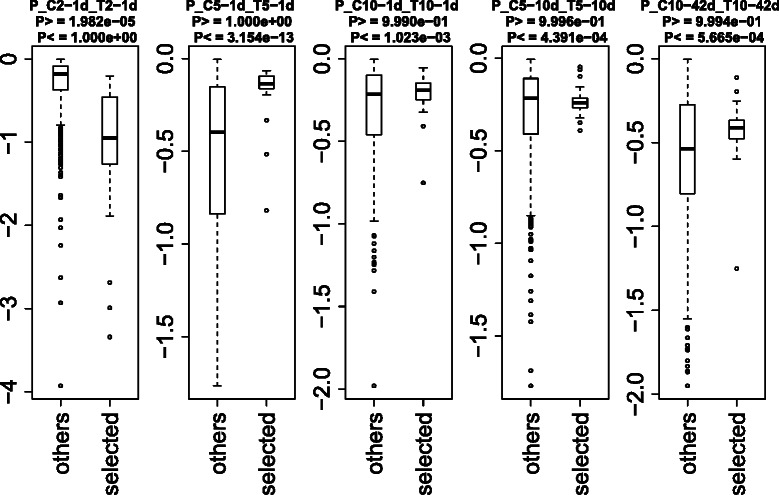
Boxplots of logarithmic *P*-values obtained from *t* tests comparing control and treatment samples.*P*-values < 0.5 indicate greater miRNA upregulation in treatment samples than controls (justification for using logarithmic *P*-values is available in Additional file 2). From left to right, the experimental conditions were 2, 5, and 10 days of stress and 1 day of rest; 5 days of stress and 10 days of rest; and 10 days of stress and 42 days of rest. Right-hand boxes (“selected”) are the 27 miRNAs identified by PCA-based unsupervised FE, and left-hand boxes (“others”) the remaining miRNAs. *P*-values shown above each plot were calculated using *t* tests to compare logarithmic *P*-values between selected and other miRNAs. *P*_>_s (*P*_<_s) is the rejection probability that rejects the null hypothesis (both sets have equal means) in favor of the alternative hypothesis (“others” have a larger or smaller mean than “selected”). Based on these criteria, selected miRNAs are upregulated in treatment samples with 2 days of stress and 1 day of rest, and downregulated in treatment samples for the other conditions.

In order to determine the effectiveness of CPCAFE in a real application, we examined PTSD associated heart disease. Our data set consisted of multiclass categorical samples, including several experimental conditions with no pre-defined rank orders (see Methods), and is suitable for estimating CPCAFE performance. Two-dimensional embedding of miRNA profiles are shown (Figure 6). Two probes (corresponding to mmu-miR-302c and -370) with extremely large values were deemed to be erroneous signals and excluded prior to embedding. Since each miRNA was attributed to multiple probes, removing these two probes did not mean exclusion of these miRNAs from the analysis. In addition, probes with relatively large PC2 projections were excluded and the 100 top-ranked outliers along PC1 selected. This strategy ensured selection of PC1 enhanced probes, as outliers along both PC1 and PC2 were not expected to be PC1 specific (PCs other than PC1 or PC2 were excluded, with our rationale discussed in Additional file 2). CPCAFE assumes that if a set of probes are outliers along a PC, they behave in a group oriented manner. If not, they are not outliers and are instead located on the origin, where the majority of probes without biological feasibility are presumed to be located. This criterion requires no user input and probe biological relevance (e.g., distinct expression between treatment and control samples) is unknown. Using these assumptions, we further investigated a selected 100 probes, representing 27 unique miRNAs, specifically, mmu-miR-451, -22, -133b, -709, -126-3p, -30c, -29a, -143, -24, -23b, -133a, -378, -30b, -29b, -125b-5p, -675-5p, -16, -26a, -30e, -1983, -691, -23a, -690, -207, and -669l, and mmu-let-7b and -7g. We investigated differential expression of these miRNAs between treatment and control samples using various statistical tests (e.g., *t*-test, Wilcoxon rank sum test, and Kolmogorov-Smirnov test), but obtained no significant results. However, owing to the small number of samples (four samples for treatment and control conditions), the significance criterion (i.e., *P*<0.05) was not satisfied by any miRNA examined, including the 27 selected miRNAs. Therefore, we compared the selected and other miRNAs as two separate groups. Boxplots of logarithmic *P*-values obtained by *t* tests between miRNAs in treatment and control samples are shown (Figure 7).

The 27 selected miRNAs have larger or smaller *P*-values than the other miRNAs, indicating that CPCAFE successfully selected differentially expressed miRNAs between treatment and control samples (justification for using logarithmic *P*-values is provided in Additional file 2).

Although the selected miRNAs were expressed distinctly from the other miRNAs, this does not demonstrate biological relevance. As our aim was to investigate the underlying transcriptomic background of PTSD-mediated heart disease, preliminary validation of our approach can be provided by prior involvement of the selected miRNAs with heart disease. To address this, we performed literature searches (Table 2). Of the 27 selected miRNAs, 23 (excluding miR-1983, -691, -690, and -207) were previously reported to be related to heart disease, suggesting our miRNA selection is biologically useful.

**Table 2:** **Summary of studies with association between the 27 selected miRNAs and heart disease**

**miRNAs**	**Ref.**	**Description**
miR-451	[39]	Upregulated in heart due to ischemia
miR-22	[40]	Elevated serum levels in patients with stablechronic systolic heart failure
miR-133	[41]	Downregulated in transverse aortic constrictionand isoproterenol-induced hypertrophy
miR-709	[42]	Upregulated in rat heart four weeks after chronicdoxorubicin treatment
miR-126	[43]	Association with outcome of ischemic andnonischemic cardiomyopathy in patients withchronic heart failure
miR-30	[44]	Inversely related to CTGF in two rodent modelsof heart disease, and human pathological leftventricular hypertrophy
miR-29	[45]	Downregulated in the heart region adjacent toan infarct
miR-143	[46]	Molecular key to switching of the vascular smoothmuscle cell phenotype that plays a critical role incardiovascular disease pathogenesis
miR-24	[47]	Regulates cardiac fibrosis after myocardial infarction
miR-23	[48]	Upregulated during cardiac hypertrophy
miR-378	[49]	Cardiac hypertrophy control
miR-125	[50]	Important regulator of hESC differentiation to cardiacmuscle(potential therapeutic application)
miR-675	[51]	Elevated in plasma of heart failure patients
let-7	[52]	Aberrant expression of let-7 members incardiovascular disease
miR-16	[53]	Circulating prognostic biomarker in critical limbischemia
miR-26	[54]	Downregulated in a rat cardiac hypertrophy model
miR-669	[55]	Prevents skeletal muscle differentiation in postnatalcardiac progenitors

To further confirm biological suitability of the identified miRNAs, we examined KEGG pathway enrichment using miRNA target genes (see Methods). Associations between enriched KEGG pathways and heart disease are summarized (Table 3). Next, we examined the 21 top-ranked pathways (the complete list is available in Additional file 3), with 17 found to be related to heart disease. Overall, these findings validate both our selection of miRNAs, and utility of CPCAFE.

**Table 3:** **Summary of studies with association between heart disease and KEGG pathways enriched by miRNA target genes**

**KEGG pathway**	**Enrichment** ***P*** **-value**	**Ref.**	**Description**
Axon guidance	3.61×10^−14^	[56]	Axon guidance of sympathetic neurons to cardiomyocytes is a promising target for regulation of cardiac function in diseased hearts
Colorectal cancer	1.92×10^−10^	[57]	Significant association between colorectal neoplasm and coronary artery disease
Chronic myeloid leukaemia	1.92×10^−10^	[58]	Imatinib mesylate (a therapeutic agent for chronic myeloid leukaemia) has cardiotoxicity
Glutamatergic synapse	3.35×10^−9^	—	—
Hepatitis B	5.86×10^−9^	[59]	Hepatitis B virus causes varied forms of heart disease
Pancreatic cancer	6.60×10^−9^	—	—
Acute myeloid leukaemia	2.54×10^−8^	[60]	Cause of acute ischemic heart disease
Focal adhesion	6.65×10^−8^	[61]	Focal adhesion kinase deletion attenuates pressure overload-induced hypertrophy
MAPK signaling pathway	3.50×10^−7^	[62]	Plays an important role in cardiac and vascular disease pathogenesis
Endometrial cancer	4.55×10^−7^	[63]	Cardiovascular disease is the leading cause of death among endometrial cancer patients
Chagas disease	6.33×10^−7^	[64]	Association with heart disease
T cell receptor signaling pathway	9.82×10^−7^	—	—
ErbB signaling pathway	1.01×10^−6^	[65]	ErbB-signaling pathway proteins are potential drug targets for heart failure treatment
Prostate cancer	2.47×10^−6^	[66]	Coronary artery disease and prostate cancer are both common diseases sharing many risk factors
Neurotrophin signaling pathway	3.08×10^−6^	—	—
Toxoplasmosis	3.16×10^−6^	[67]	Toxoplasmosis is a cause of heart disease
Bacterial invasion of epithelial cells	3.84×10^−6^	—	—
TGF-beta signaling pathway	7.35×10^−6^	[68]	Upregulated in infarcted myocardium
Nonsmall-cell lung cancer	9.00×10^−6^	—	—
VEGF signaling pathway	1.17×10^−5^	[69]	A VEGF inhibitor has cardiotoxicity
Dopaminergic synapse	2.19×10^−5^	[70]	Dopamine agonists affect the cardiovascular system

Moreover, our results suggest that the aberrantly expressed miRNAs are likely involved in PTSD-mediated heart disease. Hence, further investigation of the miRNA target genes may clarify the underlying molecular biology and transcriptomic background of PTSD-mediated heart disease.

**Figure 8:**
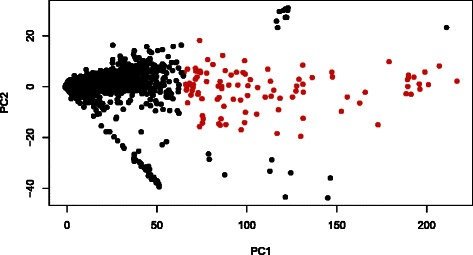
Two-dimensional embedding of mRNA expression by PCA. Notations are the same as in Figure 6. See Additional file 2 for more detail.

**Figure 9:**
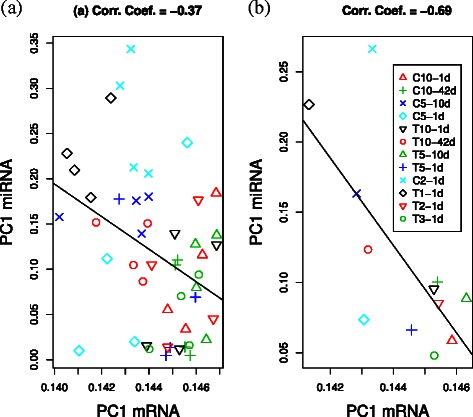
Comparison of sample contributions to PC1 between mRNA and miRNA.**(a)** Scatterplot comparing mRNA and miRNA expression profiles for sample contribution to PC1 (i.e., components of the first loading vector). Pearson’s correlation coefficient =−0.37 (*P*=0.01). **(b)** Averaged contributions within each condition. Pearson’s correlation coefficient =−0.69 (*P*=0.01). XY-Zd with X = C (control); X = T (treatment); Y, stress days; and Z, rest days in experimental conditions. See Additional file 2 for more detail.

In this regards, we applied PCA to mRNA expression. Two-dimensional embedding of mRNA expression is shown (Figure 8). To determine correlation between miRNA and mRNA expression, we compared their expression profiles and determined the contribution of each sample to PC1 (Figure 9(a)), showing negative correlation between PC1s. Additionally, to determine if the observed correlation is coincident with experimental conditions, scatterplots of averaged values within each condition were examined (Figure 9(b)). This strengthened the correlations but did not change significance, indicating that the observed negative correlation between mRNA and miRNA expression is reliable. Next, we selected mRNAs using CPCAFE. To select PC1 enhanced probes, the 100 top-ranked outliers along PC1 were selected, excluding probes with relatively large projections to PC2. In total, 59 unique mRNAs were identified (RefSeq mRNA IDs are provided in Additional file 4). To confirm negative correlation between miRNAs and miRNA target mRNAs, correlation coefficients were determined (see Additional file 4). There were no targets common to the selected 59 mRNAs and TarBase, employed by DNA intelligent Analysis (DIANA)-mirpath [11] and used in KEGG pathway analysis (see Methods), possibly because of TarBase’s experiment-oriented, thus context-dependent, nature (i.e., TarBase does not include PTSD). Thus, instead we used seed matching to identify miRNA target genes, with so called 7mer-m8 [12] detecting exact matches to positions 2-8 of mature miRNAs (seed + position 8). Among the 59 mRNAs, 24 were targeted by at least one of the 27 selected miRNAs. In addition, 47 pairs of miRNAs and miRNA target genes were identified. In total, there were 45/47 negative correlation coefficients between miRNAs and miRNA target genes. We also examined correlation coefficient significance (see Methods), with 26/47 pairs (more than half) associated with significant correlations (two positive correlations were judged insignificant), and confirming negative correlation between miRNAs and miRNA target genes.

In order to determine if the selected 59 mRNAs were differentially expressed between control and treated samples, we examined the logarithmic ratio. Using *t* tests, the averaged logarithmic ratio of the 59 samples was significantly negative or positive compared with that averaged over other mRNAs, excluding only one condition (see Table 4). Thus, our results show that the 59 selected mRNAs are mostly distinctly expressed between control and treated samples.

**Table 4:** ***P*** **-values calculated by** ***t*** **tests from logarithmic ratios between treated and control samples**

	**C2-1d**	**C5-1d**	**C10-1d**	**C5-10d**	**C10-42d**
***P*** **-values**	**vs**	**vs**	**vs**	**vs**	**vs**
	**T2-1d**	**T5-1d**	**T10-1d**	**T5-10d**	**T10-42d**
Control < treated	6.9×10^−4^	7.4×10^−8^	1.0	0.22	0.03
Control > treated	1.0	1.0	2.35×10^−8^	0.78	0.97

Smaller *P*-values indicate that the difference in mean logarithmic ratio is more significant in the selected 59 mRNAs than that in the other mRNAs. For descriptions of each experimental condition, see the caption for Figure 9. For more methodological detail, see Additional file 2.

Although the selected mRNAs include many targeted and negatively regulated by miRNAs, and are differentially expressed between control and treated samples, published associations between the selected mRNAs and heart disease would provide direct biological relevance. Association between the identified genes and heart disease are summarized (Table 5). In total, 9 genes were both targeted by at least one of the 27 miRNAs and related to heart disease. In addition, 17 genes were related to heart disease but not targeted by any of the 27 miRNAs (see Additional file 5). Among the genes not associated with heart disease, 15 genes were targeted by at least one of the 27 miRNAs, and only 18 genes were not targeted by any. Because approximately half (26 in total) of the selected 59 genes are related to heart disease, our gene selection is feasible from a biological point of view.

**Table 5:** **Summary of studies with association between heart disease and genes selected by CPCAFE**

**Gene**	**RefSeq mRNA**	**miRNAs**	**Heart disease association (** ***P*** **-values from the Gendooserver)**
*Fabp3*	NM_010174	miR-709	Cardiomegaly (0.031)
*Cox4i1*	NM_009941	miR-709	Cardiac output, low (5×10^−4^)
*Atp5g1*	NM_001161419	miR-29a/b-3p,	Cardiomyopathies (1.5×10^−3^)
		miR-16	
*Cox6a2*	NM_009943	miR-23a/b-3p	Heart disease (1.3×10^−3^); Cardiomyopathies (2.3×10^−3^);Heart failure (5.0×10^−3^)
*Aldoa*	NM_001177307	miR-16	Cardiomyopathy, dilated (2.0×10^−3^)
*Cox5a*	NM_007747	miR-26a-5p	Cardiomyopathies (5.4×10^−3^); Myocardial ischemia(7.4×10^−4^)
*Myl2*	NM_010861	miR-1983	Heart defects, congenital (4.9×10^−48^); Cardiomyopathy, dilated (5.2×10^−21^); Cardiomegaly (1.1×10^−17^); Heart failure (2.1×10^−9^)
*Myl3*	NM_010859	miR-691	Heart defects, congenital (1.1×10^−7^); Cardiomegaly(1.2×10^−4^); Myocardial infarction (2.4×10^−4^); Heart septal defects, ventricular (6.9×10^−4^)
*Tcap*	NM_011540	miR-207	Cardiomyopathy, dilated (1.1×10^−8^); Cardiomyopathy, hypertrophic (1.7×10^−3^); Heart defects, congenital(6.0×10^−3^); Heart failure (1.2×10^−2^)

Finally, we compared the selected 59 genes with KEGG pathways. KEGG pathway analysis had been performed for the miRNAs, but as they are not the only mRNA regulatory mechanism, mRNA expression may be, at least partially, distinct from miRNA expression. We found many genes were concentrated to specific KEGG pathways (see Additional file 6). For example, *Uqcrh*, *Uqcrq*, *Cox6a2*, *Cox7a1*, *Cox5a*, *Cox4i1*, *Cox8b*, *Cox6c*, *Myl2*, *Myl3*, *Myh6*, *Myh8*, *Tpm1*, *Tnni3*, and *Tnnt2* belong to the KEGG pathway “Cardiac muscle contraction” (mmu04260), and most are also components of cytochrome c oxidase, involved in mitochondrial proton transfer. Thus, it is biologically reasonable that heart disease is associated with aberrant expression of these genes. *Mybpc3*, *Tnni3*, and *Tnnt2* belong to the KEGG pathway “Hypertrophic cardiomyopathy” (mmu05410), also coincident with a role in heart disease. *Atp5g1*, *Atp5b*, *Atp5g3*, *Atp5h*, *Atp5a1*, *Atp5e*, *Atp5j2*, *Ndufa13*, and *Ndufs6* belong to “Oxidative phosphorylation” (mmu00190 + 11951). This KEGG pathway is an essential part of mitochondrial energy metabolism, and malfunction is likely to be directly related to muscle functionality. *Mdh2* and *Aco2* belong to the “Citrate cycle” (mmu00020), and are involved in energy production, while *Fabp3* belongs to the “peroxisome proliferator-activated receptor (PPAR) signaling pathway” (mmu03320), involved in muscle function. Thus, based on these KEGG pathway functions, the genes identified by CPCAFE are related to heart disease.

**Figure 10:**
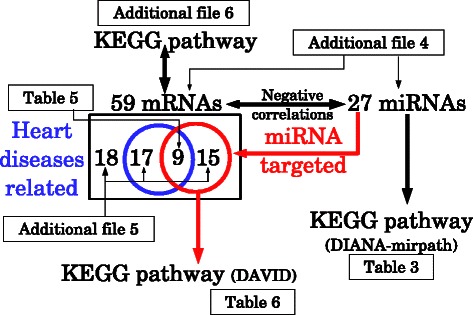
Schematic that illustrates biological validations towards 27 miRNAs and 59 mRNAs.

In order to further confirm the validity of our methodology, i.e., integrated analysis of mRNA and miRNA expression, we have also performed KEGG pathway analysis by the Database for Annotation, Visualization and Integrated Discovery (DAVID) [13] restricted to 24 genes targeted by at least one of 27 miRNAs (see Methods). DAVID identified five KEGG pathways associated with significant *P*-values adijutsted by Benjamini and Hochberg (BH) critetion (Table 6). Two out of five were related to cradiadic diseases (“Cardiac muscle contraction” and “Oxidative phosphorylation”[14]) and three were related to neurodegenerative diseases (“Parkinson’s disease”, “Alzheimer’s disease” and “Huntington’s disease”). Thus, these are very coincident with those causing PTSD mediated heart diseases. Figure 10 summarizes the analyses performed in the above.

**Table 6:** **KEGG pathway enriched by 24 mRNAs targeted by 27 miRNAs, identified by DAVID**

**KEGG pathway**	**Number of genes**	**%**	***P*** **-values**	**Adjusted** ***P*** **-values**
Cardiac muscle contraction	7	30.4	2.30E-09	3.20E-08
Parkinson’s disease	7	30.4	5.80E-08	4.10E-07
Oxidative phosphorylation	6	26.1	2.30E-06	1.10E-05
Alzheimer’s disease	6	26.1	1.20E-05	4.20E-05
Huntington’s disease	6	26.1	1.20E-05	3.50E-05

Number of genes are genes included in pathway, % is the ratio genes included in pathway among 24 genes. *P*-values and those adjusted by BH criterion were provided by DAVID.

Our findings suggest that PTSD-mediated heart disease may be caused by malfunction in “Cardiac muscle contraction” and/or “Oxidative phosphorylation” of energy metabolism. These malfunctions are related to aberrant gene expression likely mediated by aberrant miRNA expression. From a therapeutic point of view, PTSD-mediated heart disease may be treated based upon this knowledge. To perform *in silico* drug discovery for these genes, the 26 genes associated with heart disease were investigated. First, tertiary structures were predicted (see Methods), and found to be similar to predicted or experimentally determined tertiary structures available in the Protein Data Bank (PDB), indicating our tertiary structures are reliable (see Additional file 7).

Among the proteins with tertiary structures predicted or available in PDB, FABP3 has a “pocket” to which inhibitors can bind, and was therefore selected as a candidate drug target for *in silico* drug discovery. FABP3 is an acid binding protein and its function can be blocked by inhibition of acid binding. Moreover, FABP3 is upregulated in patients with ventricular-septal defects in comparison to normal controls [15]. FABP3 is also a member of FABPs that play critical roles in the PPAR signaling pathway identified above. Thus, it is likely that FABP3 is a key protein in PTSD-mediated heart disease.

The 10 top-ranked drug candidate compounds obtained by *in silico* drug discovery (see Methods) are listed (Table 7). The complete list of compounds ranked by FPAScores is available in Additional file 8. The compounds include promising drug candidates, for example, two heat shock protein 90 (HSP90) inhibitors are upregulated in dilated cardiomyopathy [16], and two PPAR inhibitors are regarded as pharmacological therapeutic targets [17]. Furthermore, overexpression of two Cyclin-dependent kinase 2 (CDK2) inhibitors results in smaller mononuclear cardiomyocytes [18]. All of these candidates should be investigated further.

**Table 7:** **Top ranked FABP3 inhibitor compounds identified by** ***in silico*** **drug discovery**

**Rank**	**FPAScore**	**Drug name**	**DrugBank/CHEMBL No.**	**Target/Activity reported in DrugBankand CHEMBL**
1	907.4	Oxaprozin	DB00991/CHEMBL1071	PTGS1 (Cox1) & PTGS2 (Cox2) inhibitor
2	901.9	—	DB08539/CHEMBL249736	Inhibits human CDK2
3	866.2	—	DB06964/CHEMBL252124	HSP90 *β* binding affinity
4	864.4	—	DB08702/—	Metallo- *β*-lactamase L1*
5	826.7	—	DB08396/—	Ig heavy chain V-III region CAM*
6	809.3	—	DB06908/CHEMBL209749	PPAR *α*/*γ* binding affinity
7	790.5	—	DB08483/CHEMBL47590	Agonist activity at human PPAR *δ*/*γ*/*α*; CDK2/4 inhibition
8	773.8	Flavoxate	DB01148/CHEMBL1493	CHRM1/2 antagonist; PDE 4/7/8 inhibitor
9	771.6	—	DB07594/CHEMBL399530	Inhibits HSP90 activity
10	761.2	Rolitetracycline	DB01301/CHEMBL1237046	Inhibitor of 30S ribosomal protein S9 & 16S rRNA; Inhibits synthetic amyloid *β*-42 fibrillization

*pharmacological action unknown.

### Stability and comparison with other methods

We have shown biological feasibility of CPCAFE when applied to PTSD. However, it is also important to compare its performance against other methods, as CPCAFE superiority is doubtful if any other method can achieve comparable performance. Hence, we used a modified data set to ensure performance is due to the method and not the data set. If slight modifications of the sample data set drastically decrease performance, CPCAFE superiority is doubtful. In addition, VBPCAFE performance on the PTSD data set is unknown. If VBPCAFE derives completely different outcomes from CPCAFE, proposing CPCAFE as an alternative to VBPCAFE (with a firmer theoretical base, albeit a more time-consuming method) would be less convincing.

**Figure 11:**
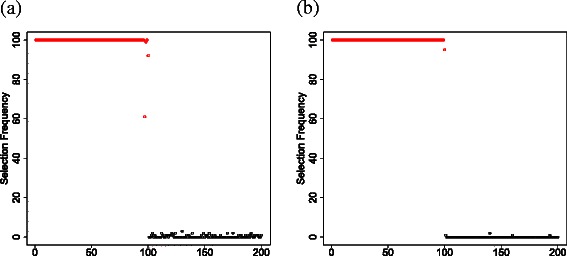
Frequency of 100 probes selected by CPCAFE and also identified by VBPCAFE within the 100 top-ranked probes with largest $C_{B}^{i1}$ values over 100 independent ensembles.**(a)** miRNA; and **(b)** mRNA. Red and black circles correspond to probes selected or not selected, respectively, by CPCAFE.

**Figure 12:**
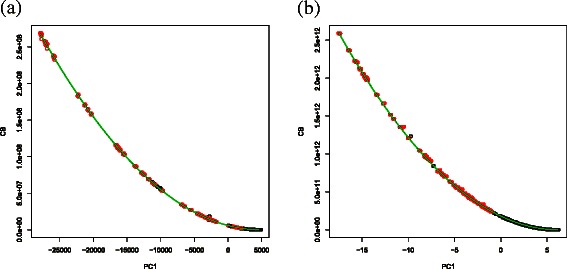
Scatterplots between *B*_*i*1_ (horizontal axis) and $C_{B}^{i1}$ (vertical axis). Red and black open circles correspond to 100 features selected or not selected, respectively, by CPCAFE. Solid green lines indicate quadratic regression lines. **(a)** miRNA; and **(b)** mRNA.

First, we examined equivalence between CPCAFE and VBPCAFE. VBPCAFE is too time-consuming to be directly applied to the whole PTSD data set, therefore we created a data set small enough to be used directly that consisted of 200 features, with 100 features selected by CPCAFE and 100 distinct features. If VBPCAFE and CPCAFE are equivalent, the 100 features selected by CPCAFE should have larger $C^{i1}_{B}$s than those attributed to the other 100 features (see Methods). Feature frequencies selected from the 100 top-ranked probes with larger $C^{i1}_{B}$ values after investigation of 100 independent ensembles are shown (Figure 11). Scatterplots between $C_{B}^{i1}$ and *B*_*i*1_ are also shown (Figure 12). As expected (from eq. (1) in Methods), $C_{B}^{i1}$ is quadratically dependent upon *B*_*i*1_, definitely demonstrating equivalence between CPCAFE and VBPCAFE when applied to a real data set. Of course, there is still the possibility that VBPCAFE applied to the whole PTSD data set would select a completely different set of features from the 100 features selected by CPCAFE. However, we believe it is unlikely as there are almost no features not selected by CPCAFE, within the 100 top-ranked in any of the 100 ensembles. Thus, CPCAFE and VBPCAFE show equivalence when applied to not only simulated data, but also a real data set.

Next, we examined CPCAFE stability, i.e., sample independence, by performing a 4-fold cross-validation study (see Methods). We specifically used a 4-fold cross-validation as there are only four biological replicates in each experimental condition, therefore using any other cross-validation would not be straightforward. The results from 100 independent ensembles are shown (see Additional file 9). For mRNAs, 78 probes were always selected by CPCAFE and only 10 probes not always selected, demonstrating high sample independence. Among the 78 probes always selected, 53 had associated RefSeq IDs that were part of the 59 RefSeq IDs selected by applying CPCAFE to the whole data set. For miRNAs, 27 probes were always selected and no probe not always selected (see Additional file 9). Among the 27 selected mature miRNAs, 25 were part of the 27 mature miRNAs selected by CPCAFE previously. Thus, CPCAFE selected almost all features 100% of the time, demonstrating stability and suggesting that performance is not likely due to the selected data set. As CPCAFE and VBPCAFE show potential equivalence, it is likely that VBPCAFE also shares this stability.

Third, we determined if other conventional supervised, and thus possibly sample dependent, FE methods can achieve a comparable performance to CPCAFE. As there are as many as 12 experimental setups without any pre-defined rankings or orderings, it is not straightforward to use a popular multiclass oriented FE with regression analyses, e.g., lasso [1]. Not only is there no way to attribute labels with actual values to each experimental setup, but it is also not known if it is possible, in principal, to align these 12 experimental conditions in one dimensional order. Thus, the number of usable supervised FE methods that can be tested on the present PTSD data set is limited. Nevertheless, we identified and examined two methods. The first was regression analysis, which attributes a 0 or 1 to each experimental setup with a linear combination assumed to represent mRNA/miRNA expression (categorical regression-based FE, see Methods). Each feature is then ranked by significance (small *P*-values are attributed to regression), and the 100 top-ranked features selected. Using this method, we identified 100 mRNA/miRNA probes and investigated biological feasibility and stability of selected ones. For mRNAs, among the 100 selected probes, only 23 had associated RefSeq IDs (see Additional file 4), a smaller number than CPCAFE, suggesting that CPCAFE more readily identifies biologically important genes (with the underlying assumption that biologically important genes are more likely to have RefSeq IDs). Disease association of the selected genes was also poorer than those selected by CPCAFE (Table 8). Only 4/23 genes (*Ttn, Ubr2, Gata5, and Hs2st1*) were associated with heart failure-related diseases, in contrast with the 26 heart disease associated genes (out of 59 genes) selected by CPCAFE. Even extending the target species to human, only one additional gene (*Cxc11*) was identified. Altogether, these results show that CPCAFE has greater power for identifying biologically feasible genes. For miRNAs, among the 100 selected probes, 77 were identified with unique mature miRNA names (see Additional file 4), a much larger number than CPCAFE. Again, this demonstrates the biological feasibility of CPCAFE, as each miRNA is attributed to multiple probes and should therefore have been selected simultaneously. Thus with miRNAs, a smaller number of identified unique mature miRNA names reflects more plausible FE. The identified miRNAs were uploaded to DIANA-mirpath for KEGG pathway analysis (see Additional file 3), and 74 KEGG pathways identified (66 with CPCAFE), although the number of miRNAs related to each pathway is much smaller: using CPCAFE, on average seven miRNAs contributed to each pathway, while with categorical regression-based FE, only four miRNAs contributed, despite almost three times larger (77 vs 27) miRNAs uploaded (*P* = 1.67×10^−17^, difference between mean values computed by *t* test). This suggests that categorical regression-based FE is less able to identify biologically important miRNAs than CPCAFE. We also determined if miRNA target mRNAs were negatively correlated with miRNA expression, and identified 180 mRNA-miRNA pairs but with only 35 pairs negatively correlated (see Additional file 4). This again contrasts with CPCAFE, with almost all mRNA-miRNA pairs negatively correlated. Correlation coefficient significance was examined, and only 20 significantly correlated pairs were identified. Since all significant correlation coefficients were negative, categorical correlation-based FE can extract correct features, but is less able to confidently screen more features than CPCAFE; more than half of the pairs identified by CPCAFE were associated with significant negative correlations. This is consistent with the limited number of miRNAs selected by categorical regression-based FE contributing to KEGG pathway analysis. Stability was also compared with CPCAFE (see Additional file 9). For mRNAs, only 24 probes were always selected among 100 cross-validation ensembles, with 122 probes selected only once. For miRNAs, only eight probes were always selected (see Additional file 9). The next highest frequencies selected were 93 and 95, corresponding to only two probes each. Conversely, 33 and 29 probes were selected only once and twice, respectively. Thus, with regards to stability, CPCAFE outperforms FE with categorical regression analysis.

**Table 8:** **Disease association of mRNAs selected by categorical regression-based FE and BAHSIC**

**RefSeq mRNA**	**Gene**	**Heart disease association (** ***P*** **-values: mouse, human)**
	**Categorical regression-based FE**	
NM_011652	*Ttn*	Cardiomyopathy, dilated (5.49×10^−7^, 1.80×10^−10^); Cardiomyopathies (1.5×10^−4^, 9.19×10^−5^); Cardiomyopathy, hypertrophic (5.6×10^−3^, 4.74×10^−10^); Heart defects, congenital (1.90×10^−2^, —); Heart failure (—, 1.02×10^−5^); Cardiomyopathy, hypertrophic, familial (—, 1.02×10^−5^)
NM_019494	*Cxcl11*	Cardiomyopathy, hypertrophic (—, 3.05×10^−2^)
NM_001177374	*Ubr2*	Heart Defects, congenital (8.64×10^−4^, —); Cardiomegaly (1.46×10^−3^, —)
NM_008093	*Gata5*	Heart defects, congenital (1.40×10^−7^, —); Heart septal defects (4.12×10^−3^, —); Hypertrophy, right ventricular (1.36×10^−4^, —); Cardiovascular abnormalities (1.49×10^−3^, —); Cardiomyopathy, dilated (8.14×10^−3^, —); Cardiomyopathies (9.27×10^−3^, —); Cardiomegaly (1.75×10^−2^, 7.60×10^−3^)
NM_011828	*Hs2st1*	Cardiomyopathies (3.87×10^−3^, —);
**BAHSIC**		
NM_009722	*Atp2a2*	Cardiomegaly (7×10^−21^, 1.33×10^−3^); Cardiomyopathy, dilated (2.68^−12^, 7.37^−9^); Cardiomyopathies (5.87×10^−12^, 9.69 × 10^−4^); Heart Failure (6.50 × 10^−12^, —); Cardiac Output, low (2.01 × 10^−7^, —); Arrhythmias, cardiac (8.85 ×10^−7^, —); Hypertrophy, left ventricular (3.03×10^−6^, 3.38×10^−2^); Myocardial reperfusion injury (2.33×10^−5^, —) Myocardial stunning (3.42×10^−3^, —); Atrial fibrillation (5.42×10^−3^, —); Myocardial infarction (6.11×10^−3^, —); Heart disease (2.38×10^−2^, 7.00×10^−4^); Ventricular dysfunction, left (2.74×10^−2^, 3.4×10^−4^); Cardiomyopathy, restrictive (—, 2.12×10^−3^); Myocardial ischemia (—, 2.91×10^−3^); Mitral valve insufficiency (—, 3.68×10^−3^); Cardiomyopathy, hypertrophic (—, 3.49×10^−2^)
NM_001164171	*Myh6*	(Already identified by CPCAFE)
NM_008725	*Nppa*	Heart Defects, congenital (2.53×10^−55^, 9.09×10^−9^); Cardiomegaly (1.11×10^−28^, —); Cardiomyopathies (7,57×10^−17^, —); Hypertrophy, left ventricular (9.16×10^−12^, 1.19×10^−13^); Cardiomyopathy, dilated (6.47×10^−11^, 2.41×10^−8^); Heart disease (9,72×10^−8^, 1.11×10^−4^); Endocardial cushion defects (3.51×10^−6^, —); Heart septal defects (4.63×10^−6^, —); Cardiovascular abnormalities (6.34×10^−5^, —); Tachycardia, ectopic atrial (1.97×10^−4^, —); Arrhythmias, cardiac (4.35×10^−4^, —); Cardiomyopathy, hypertrophic, familial (4.90×10^−3^, —); Heart septal defects, ventricular (5.70×10^−3^, —); Hypertrophy, right ventricular (1.04×10^−2^, —); Heart failure (1.04×10^−2^, 5.07×10^−29^); Cardiomyopathy, hypertrophic (2.20×10^−2^, 4.40×10^−2^) Ventricular dysfunction, left (—, 3.04×10^−10^); Myocardial reperfusion injury (— 1.76×10^−7^); Cardiovascular disease (—, 4.21×10^−6^); Myocardial infarction (—, 9.73×10^−6^); Mitral valve insufficiency (—, 1.04×10^−5^); Heart valve disease (—, 3.27×10^−5^); Myocardial ischemia (—, 1.49×10^−4^); Ventricular dysfunction (—,.198×10^−3^); Cardiomyopathy, restrictive (—, 2.68×10^−2^); Ventricular dysfunction, right (—, 3.53×10^−3^)
NM_008103	*Gcm1*	Heart defects, congenital (6×10^−3^, —)
NM_009608	*Actc1*	Heart defects, congenital (2.54×10^−38^, —); Cardiomyopathy, dilated (6.55×10^−9^, 2.16×10^−14^); Heart septal defects (5.15×10^−7^, 6.80×10^−4^); Arrhythmias, cardiac (4.90×10^−5^, —); Cardiomyopathies (2.75×10^−4^, 1.23×10^−2^); Heart septal defects, atrial (1.33×10^−3^); Cardiovascular abnormalities (3.85×10^−2^, —); Cardiomegaly (4.45×10^−2^, 1.01×10^−4^); Cardiomyopathy, hypertrophic (—, 4.91×10^−13^); Cardiomyopathy, hypertrophic, familial (—, 1.97×10^−6^); Cardiomyopathy, restrictive (—, 5.87×10^−4^); Heart septal defects, atrial (—, 1.73×10^−3^); Hypertrophy, left ventricular (—, 1.09×10^−2^)
NM_013468	*Ankrd1*	Heart defects, congenital (3.98×10^−12^, 4.70×10^−5^); Cardiomegaly (2.52×10^−6^, 1.00×10^−2^); Cardiomyopathies (9.11×10^−5^, —); Heart septal defects (6.18×10^−4^, —); Cardiomyopathy, hypertrophic, familial (9.66×10^−4^, —); Cardiomyopathy, dilated (1.22×10^−2^, 1.22×10^−2^); Heart failure (—, 3.04×10^−4^); Hypertrophy, left ventricular (—, 7.55×10^−3^)
NM_011540	*Tcap*	(Already identified by CPCAFE)
NM_177369	*Myh8*	(Already identified by CPCAFE)
NM_007450	*Slc25a4*	(Already identified by CPCAFE)
NM_010174	*Fabp3*	(Already identified by CPCAFE)
NM_008084	*Gapdh*	(Already identified by CPCAFE)
NM_019494	*Cxcl11*	Already identified by categorical regression based FE
NM_013463	*Gla*	Cardiomyopathy, hypertrophic (—, 2.46×10^−2^); Heart disease (—, 2.64×10^−2^); Cardiomyopathies (—, 3.10×10^−2^)
NM_001038592	*Glrx2*	Myocardial reperfusion injury (—, 1.56×10^−3^)

*P*-values obtained from the Gendoo server for both mouse and human. Genes annotated as “Already identified by CPCAFE” are listed in Table 5.

It is possible that CPCAFE outperforms FE with categorical regression analysis because it is too simple (naive) an alternative. Therefore to compare CPCAFE performance with a more sophisticated method, we used BAHSIC (see Methods).

**Figure 13:**
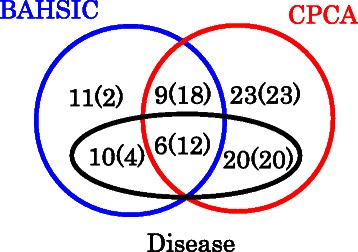
Venn diagram of mRNAs extracted by CPCAFE and BAHSIC, and heart failure-related disease association. Numbers in parentheses correspond to those with outliers along PC2 (see Additional file 2).

For mRNAs, among the 100 selected probes, only 37 had RefSeq IDs, again less than CPCAFE (see Additional file 4). Of these 37 mRNAs, only 16 were associated with heart failure-related disease (Table 8), even when the target species was extended to human. Thus, 43% of extracted RefSeq associated mRNAs are related to heart disease in the Gendoo server. This is similar to the 45% reported for genes selected by CPCAFE. Considering that the total number of selected RefSeq associated mRNAs is larger, this suggests that CPCAFE outperforms BAHSIC as well as categorical regression based FE. Interestingly, 15/37 mRNAs (not always including the 16 disease associated genes) were also selected by CPCAFE, despite use of distinct algorithms by BAHSIC and CPCAFE. Nine genes selected by CPCAFE (in addition to the six genes listed in Table 8 or Additional file 5), but with missing disease association were *Synrg, Milr1, Exosc2, Medag, 2610028H24Rik, pbld1, Hbb-bt, 1700047I17Rik2*, and *Hba-a2*. Thus, mRNAs extracted by CPCAFE and BAHSIC overlap significantly. Furthermore, if we also consider PC2 outliers (see Additional file 2) that were excluded in the previous analyses, an additional 15 genes (*Rhox8, Kctd14, Ttc38*, *G* *l* *r* *x*2, *Ndor1, Dcc*, *E* *l* *k*1, *G* *c* *m*1, *Hccs*, *Nppa*, *A* *c* *t* *c*1, *Pigr, Slc10a1*, *C* *x* *c* *l*11, and *Med16*) have been identified by CPCAFE (Bolditalic six genes are also associated with heart failure-related disease, see Additional file 2). Consequently, there is a substantial increase in overlap significance between mRNAs extracted by CPCAFE and BAHSIC. Figure 13 shows the relationship between CPCAFE, BAHSIC, and heart failure-related disease association. The many genes identified by both methods and associated with disease show that our method (i.e., treating expression data as a categorical multiclass data set and not a collection of pairwise comparisons) can identify biologically relevant genes.

For miRNAs, among the 100 probes, 47 unique mature miRNAs were selected (see Additional file 4), a much larger number than CPCAFE (27). The 47 miRNAs were uploaded to DIANA-mirpath (see Additional file 4), and 65 KEGG pathways identified as significant. Although this number is similar to that obtained by CPCAFE (66), considering that the number of uploaded miRNAs was almost twice (47 vs 27), CPCAFE is still superior to BAHSIC. In fact, the averaged number of miRNAs with target mRNAs enriched in each pathway, was approximately seven, similar to that obtained by CPCAFE despite two-times more uploaded miRNAs. We also compared biological significance of the KEGG pathways obtained by BAHSIC and CPCAFE. In contrast to 17 reported pathways related to heart-failure related disease (from the top most significant 21 KEGG pathways) using CPCAFE (Table 3), only 11 identified by BASHSIC (of which, seven were also identified by CPCAFE; Table 3) were related to heart-failure related disease by literature searching (Table 9). Thus, from a biological point of view, CPCAFE outperforms BAHSIC. We also determined if targeted mRNAs negatively correlate with miRNA expression, and identified 164 mRNA-miRNA pairs, although only 73 were negatively correlated (see Additional file 4). Conversely, with CPCAFE, almost all pairs are negatively correlated. Correlation coefficient significance was examined, with only 33 significantly correlated pairs identified. Since all significant correlation coefficients were negative, this shows that BAHSIC cannot correctly extract features, and is less able to confidently screen more features than CPCAFE. Again this coincides with the limited number of miRNAs selected by BAHSIC that contribute to KEGG pathway analysis.

**Table 9:** **Summary of studies with association between heart disease and KEGG pathways enriched by BAHSIC identified miRNA target genes**

**KEGG pathway**	**Enrichment** ***P*** **-value**	**Ref.**	**Description**
Long-term depression	5.37×10^−19^	[71]	Depression has been linked with additional health problems, including heart disease
Prion diseases	3.13×10^−17^	[72]	Prion-induced amyloid heart disease with high blood infectivity in transgenic mice
Axon guidance	5.99×10^−14^	(Already identified by CPCAFE)	
Fatty acid biosynthesis	2.01×10^−12^	[73]	A relationship between fatty acid synthase andcardiac calcium signaling has been suggested
Calcium signaling pathway	2.04×10^−10^	(Already identified by CPCAFE)	
Gap junction	5.03×10^−10^	[74]	Gap junction alterations in human cardiac disease
Prostate cancer	6.04×10^−10^	(Already identified by CPCAFE)	
Pancreatic cancer	1.63×10^−9^	—	—
Glutamatergic synapse	2.02×10^−8^	—	—
Endometrial cancer	2.02×10^−8^	(Already identified by CPCAFE)	
Long-term potentiation	2.65×10^−8^	—	—
Neurotrophin signaling pathway	2.81×10^−8^	—	—
Focal adhesion	8.34×10^−8^	(Already identified by CPCAFE)	
TGF-beta signaling pathway	2.00×10^−7^	(Already identified by CPCAFE)	
Endocytosis	2.27×10^−7^	—	—
Cholinergic synapse	9.60×10^−7^	—	—
Regulation of actin cytoskeleton	1.33×10^−6^	—	—
Colorectal cancer	3.52×10^−6^	(Already identified by CPCAFE)	
Salmonella infection	5.65×10^−6^	—	—
PI3K-Akt signaling pathway	1.01×10^−5^	—	—

Pathways annotated as “Already identified as CPCAFE” are listed in Table 3.

BAHSIC stability was compared (see Additional file 9). For mRNAs, only one probe was selected only 14 times among the 100 independent ensembles, and was also the most frequently selected. The second most frequent four probes were selected only 13 times, and 2133 probes were selected only once. For miRNAs, 68 probes were always selected among 100 cross-validation ensembles (see Additional file 9). The second most frequent probes were selected 99 times, but this only included three probes. The third, fourth, and fifth frequent probes were selected 98, 97, and 96 times, but included only 2, 3 and 1 probes, respectively. Thus, with stability, CPCAFE definitely outperforms BAHSIC.

In conclusion, CPCAFE outperformed two conventional supervised FE methods on both stability and biological feasibility, and performed equally well to VBPCAFE, which has more theoretical confidence. Thus, CPCAFE and VBPCAFE are the most suitable FE methods for categorical multiclass problems.

**Figure 14:**
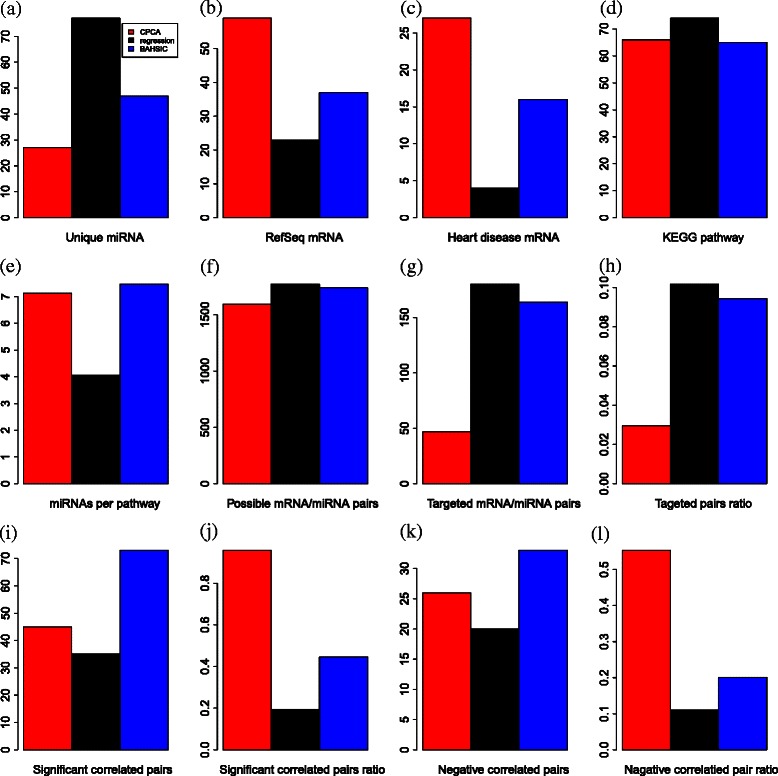
Barplots comparing CPCAFE, categorical regression based FE, and BAHSIC. See text for details about panels included, from **(a)** to **(l)**.

**Figure 15:**
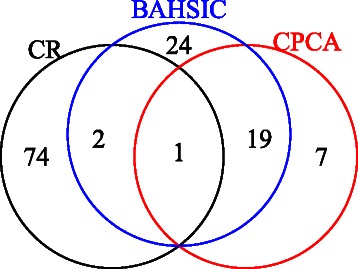
Venn diagram of miRNAs extracted by CPCAFE and BAHSIC.

We have included diagrams summarizing the discussed points (Figure 14). Figure 14(a) shows the number of unique miRNAs. The same 100 probes were extracted using all methods, and miRNAs assigned to multiple probes. As all probes to which the same miRNAs are assigned should be extracted at the same time, smaller numbers of unique miRNAs assigned to 100 probes shows better extraction. In this context, CPCAFE outperformed the other two methods (Venn diagram is also available in Figure 15). Figure 14(b) shows the number of mRNAs with RefSeq IDs. As mRNAs with RefSeq IDs are more likely to have known biological significance, a larger number of RefSeq IDs accompanying mRNAs shows selection of more biologically relevant mRNAs. In this context, CPCAFE outperformed the other two methods. Figure 14(c) shows the number of RefSeq miRNA genes related to heart failure-related disease. Larger numbers suggest that FE has correctly extracted mRNAs related to the target disease, therefore CPCAFE outperformed the other two methods. Figure 14(d) shows the number of KEGG pathways identified by uploading the miRNAs shown in Figure 14(a) to DIANA-mirpath. Although categorical regression based FE outperformed the other two methods, it may have incorrectly extracted the most unique miRNAs in Figure 14(a), therefore increased number of KEGG pathways does not always reflect method superiority. Indeed, counting the average number of miRNAs contributing to each pathway (Figure 14(e)), indicates that CPCAFE and BAHSIC are comparable while categorical regression based FE performs worse. This suggests that categorical regression based FE cannot identify as many KEGG pathways as CPCAFE or BAHSIC if the same number of unique miRNAs are extracted (albeit experimentally). Thus, it is reasonable to assume that CPCAFE and BAHSIC outperformed categorical regression based FE in this context. Figure 14(f) shows the possible number of mRNA/miRNAs pairs, i.e., product of the unique miRNAs (Figure 14(a)) and RefSeq accompanying mRNAs (Figure 14(b)). Although these numbers are comparable among the three FE methods, the number of miRNA and targeted mRNA pairs is distinct (Figure 14(g)). Considering the ratio (Figure 14(h)) (obtained by dividing the numbers in Fig. 14(g) by those in Figure 14(f)), CPCAFE appears worst (i.e., smallest), but this is reversed if significance is considered. Figure 14(i) shows the number of miRNA and targeted mRNA pairs with significant correlations. Converting these numbers to a ratio (Figure 14(j)) by dividing the number of pairs of miRNAs and targeted mRNAs (Figure 14(g)), CPCAFE outperformed the other two methods. The same result is obtained when considering negative correlations (Figures 14(k) and (l)), which are desirable as miRNAs suppress target mRNA expression. Overall, these results (from Figure 14(g)-(l)) suggest that CPCAFE extracted significantly more miRNA and targeted mRNA pairs than the other two methods, which had apparently extracted more. In conclusion, based on our summarized discussions, we conclude that CPCAFE outperforms two other FE methods.

## Conclusions

We have extended VBPCA for FE. Although VBPCAFE is an effective method when applied to simulated data, feature-dependent extension inevitably makes it computationally challenging. Thus, we replaced VBPCA with the simpler CPCA, and achieved reasonable FE performance on simulated data. In order to demonstrate CPCAFE effectiveness on real data, we performed an integrated analysis of mRNA and miRNA expression from stressed mouse heart, investigating the underlying molecular biology and transcriptomic background of PTSD-mediated heart disease. CPCAFE successfully identified aberrantly expressed miRNAs and their negatively regulated target mRNAs. Biological significance of the identified miRNAs and mRNAs was confirmed. Equivalence between CPCAFE and VBPCAFE was demonstrated by applying both methods to the PTSD data set. Two conventional supervised FE methods were also tested, with CPCAFE outperforming them both. *In silico* drug discovery was performed for a selected gene, FABP3, with the top-ranked compounds identified including protein inhibitors reportedly upregulated in heart disease.

## Methods

### Simulations based on synthetic data of categorical multiclass samples

To simulate multiclass samples with *N* features and *M* samples, expression values were modeled using Gaussian distributions with *μ*_*k*_ mean (*k* class). Standard deviations were fixed at 0.5. *μ*_*k*_ was

$$\mu_{k} = \left (\frac{k-1}{K-1} - \frac{1}{2} \right) s, k=1,\ldots,K, $$

with *K* indicating class number. Considering *x*_*ij*_ to be an expression of the *i*th feature of the *j*th sample, it obeys:

$${} x_{ij} \in \!\left \{\! \begin{array}{lcl} {\cal N}\left(\mu_{k},\frac{1}{2}\right), &1 \leq i \leq \frac{N'}{2}, & (k-1)\frac{M}{K} < j \leq k\frac{M}{K} \\ {\cal N}\left(-\mu_{k},\frac{1}{2}\right), &\frac{N'}{2} < i \leq N', & (k-1)\frac{M}{K} < j \leq k\frac{M}{K} \\ {\mathcal N}\left(\epsilon, \frac{1}{2}\right), & N' <i \leq N \end{array} \right.,$$

where ${\mathcal N} (\mu,\sigma)$ is the normal distribution with a mean of *μ* and standard deviation of *σ*. The reason for including positive and negative *μ*_*k*_ signs was to simulate coexistence of up/downregulated genes among multiple classes. The *s* parameter represents difficulty of distinguishing among multiple classes. Larger (smaller) *s* indicates easier (harder) samples with fixed *K*. *ε* is a random number satisfying the probability, *P*:

$$P(\epsilon =\mu_{k}) = \frac{1}{K} $$

In the present study, *N*=100,*N* ^′^=10,*K*=4, and *M*=20 were used, and performances averaged over 100 independent ensembles.

### One vs one *t* test based FE of a simulated categorical multiclass data set

*P* values, $P^{k,k'}_{i}$, attributed to each *i* were computed using *t* tests to compare between $\left \{ x_{\textit {ij}}; (k-1)\frac {M}{K} < j \leq k\frac {M}{K}\right \}$ and $\left \{ x_{\textit {ij}}; (k'-1)\frac {M}{K} < j \leq k'\frac {M}{K}\right \}$, for all *K*(*K*−1)/2 pairs of *k* and *k* ^′^. $P^{k,k'}_{i}$ was adjusted by BH criterion [19], with each *k*,*k* ^′^ pair and the *i*th gene adjusted. $P^{k,k'}_{i}$s <0.05 for all (*k*,*k* ^′^) pairs were identified as distinct features between multiple classes.

### Categorical regression-based FE

Categorical regression-based FE was defined as follows, *x*_*ij*_ reflects “expression” of the *j*th feature of the *i*th samples, therefore *x*_*ij*_ can be represented as:

$$x_{ij} = C_{i0} + \sum_{k} C_{ik} \delta_{jk},$$

with *δ*_*jk*_ = 1 only when the *j*th sample belongs to the *k*th category, otherwise it is 0. Category summation was taken and *C*_*ik*_s are fitting parameters. Because independent variables are categorical, the above regression equation belongs to a category of equations often named as categorical regression. For each *i*th feature, *P*-values were computed using the lm function implemented in R[20] (this can be easily performed if factors corresponding to experimental setups are used as independent variables in lm).

FE was used for simulated categorical multiclass regression in two ways. The first selected features with BH criterion [19] adjusted *P*-values <0.05. The second selected the top 10 features with smallest *P*-values. Finally, for the PTSD data set, the 100 top-ranked features with smallest *P*-values were selected as extracted features.

### BAHSIC

BAHSIC uses the Hilbert-Schmidt norm of the cross-covariance operator (HSIC), defined as follows:

$$\begin{array}{@{}rcl@{}} ||C_{jk}||^{2}_{HS} &\equiv& \left \langle \left (\sum_{i} x_{ij}x_{ij^{\prime}} \right) \delta_{jk}\delta_{j^{\prime}k^{\prime}}\delta_{kk^{\prime}} \right \rangle_{jj^{\prime}} \\ & + & \left \langle \sum_{i} x_{ij}x_{ij^{\prime}} \right \rangle_{jj^{\prime}} \left \langle \delta_{jk}\delta_{j^{\prime}k^{\prime}}\delta_{kk'} \right \rangle_{jj^{\prime}} \\ & - & 2 \left \langle \left \langle \sum_{i} x_{ij}x_{ij^{\prime\prime}} \right \rangle_{j^{\prime\prime}} \left \langle \delta_{j^{\prime}k}\delta_{j^{\prime\prime\prime}k^{\prime}}\delta_{kk^{\prime}} \right \rangle_{j^{\prime\prime\prime}} \right \rangle_{jj^{\prime}}, \end{array} $$

where 〈·〉_*j*_ and $\langle \cdot \rangle _{jj^{\prime }}$ are averaged over all *j* and *j*,*j* ^′^ pairs, respectively, and $\delta _{kk^{\prime }}$ is cronecker’s delta. The reason why linear kernel was employed was because it was shown to achieve best performances when applied to microarray gene expression [10]. In BAHSIC, features with smaller HSIC are iteratively discarded until the desired number of features remain. The number of features discarded at each step was 1 for the simulated categorical multiclass data set and 10% of remaining features for the PTSD data set.

R code for this algorithm is available in Additional file 2.

### Extended VBPCA

Before extending VBPCA, conventional VBPCA is briefly explained.

*X*={*x*_*ij*_} was modeled [2,3] as:

$$X = B A^{T} + E, $$

where *B* and *A* are *N*×*Q* and *M*×*Q* matrices, respectively, and *E* is a *N*×*M* matrix obeying a Gaussian distribution with zero mean and standard deviation, *σ*_*E*_. The purpose of VBPCA is to obtain an optimal approximation using *Q*≪*N*,*M*.

Thus, following conventional notation and after substituting *X*=*V*, the conditional probability is:

$$p(V|A,B) \propto \exp \left (-\frac{1}{{\sigma_{E}^{2}}} || V- BA^{T}||_{\text{Fro}}^{2} \right), $$

where ||·||_Fro_ is the Frobenius norm matrix. In order to obtain optimal *Q* values, a prior distribution was assumed:

$$\begin{array}{@{}rcl@{}} P(A) & \propto & \exp \left[ - \frac{1}{2} \text{tr} \left(A C_{A}^{-1} A^{T} \right)\right ]\\ P(B) & \propto & \exp \left [ - \frac{1}{2} \text{tr} \left (B C_{B}^{-1} B^{T} \right) \right], \end{array} $$

where *C*_*A*_ and *C*_*B*_ are *Q*×*Q* diagonal positive matrices *q*th (*q*=1,…,*Q*) is a diagonal element of *C*_*A*_ and *C*_*B*_ that expresses the importance of the obtained *q*th principal component. Free energy was minimized as:

$$\begin{array}{@{}rcl@{}} F &\,=\, &\frac{||V||_{\text{Fro}}^{2}}{2{\sigma_{E}^{2}}} + \frac{NM}{2} \log {\sigma_{E}^{2}} + \frac{M}{2} \log \frac{|C_{A}|}{|\Sigma_{A}|} + \frac{N}{2} \log \frac{|C_{B}|}{|\Sigma_{B}|} \\ ({C_{B}^{i}})^{-1} & + & \frac{1}{2} \text{tr} \left \{ C_{A}^{-1} \left (\hat{A}^{T}\hat{A} + M \Sigma_{A} \right) + C_{B}^{-1} \left (\hat{B}^{T}\hat{B} + N \Sigma_{B} \right) \right. \\ &\,+\,&\! \left.\sigma_{E}^{-2} \left (- 2 \hat{A}^{T} V^{T} \hat{B}\! + \left(\hat{A}^{T}\hat{A} +\! M \sigma_{A} \right)\left(\hat{B}^{T}\hat{B}\! + N \Sigma_{B} \right) \right) \right \} \\ &\,+\,& \text{const.} \end{array} $$

where *Σ*_*A*_ and *Σ*_*B*_ are variance matrices of $\hat {A}$ and $\hat {B}$, estimated from *A* and *B* by the variational method. Locally optimal $\hat {A}, \hat {B}, \Sigma _{A}, \Sigma _{B}, C_{B}$, and *σ*_*E*_ were obtained by performing the following iterative updates in this order:

$$\begin{array}{@{}rcl@{}} \Sigma_{A} &\leftarrow& {\sigma_{E}^{2}} \left(\hat{B}^{T} \hat{B} + N \Sigma_{B} + {\sigma_{E}^{2}} C_{A}^{-1} \right)^{-1}  \\ \hat{A} & \leftarrow & V^{T} \hat{B} \frac{\Sigma_{A}}{{\sigma_{E}^{2}}} \\ \Sigma_{B} & \leftarrow & {\sigma_{E}^{2}} \left (\hat{A}^{T} \hat{A} + M \Sigma_{A} + {\sigma_{E}^{2}} C_{B}^{-1} \right)^{-1}, \\ \hat{\boldmath{\text{B}}} & \leftarrow & \boldmath{\text{V}} \hat{A} \frac{\Sigma_{B} }{{\sigma_{E}^{2}}}, \\ (\Sigma_{A})_{qq}, C_{B}^{q} & \leftarrow &\frac{||\boldmath{\hat{\text{B}}}_{q}||^{2}}{N} + (\Sigma_{B})_{qq}, q=1,\ldots,Q, \\ {\sigma_{E}^{2}} & \leftarrow & \frac{1}{NM} \left \{ ||V||_{\text{Fro}}^{2} - \text{tr} \left(2V^{T}\hat{B}\hat{A}^{T} \right) \right. \\ &+& \left. \text{tr} \left (\left(\hat{A}^{T} \hat{A} + M \Sigma_{A} \right) \left(\hat{B}^{T}\hat{B} + N \Sigma_{B} \right)\right)\right\} , \end{array} $$

where $\boldmath {\hat {\text {\textit {B}}}}_{q}$ is the *q*th columnar vector of matrix $\hat {B}$, and ${C_{B}^{q}}$ is the *q*th diagonal element of *C*_*B*_. *i*th row *V* vector. In addition to the above iteration, which should be repeated from top to bottom, i.e., *Σ*_*A*_ in the left hand side of the first equation substituted to *Σ*_*A*_ in the right hand side of the second equation, *C*_*A*_ should be *I* in order to simulate conventional PCA [21]. It is also known that *C*_*B*_ can be used to estimate the optimal number of PCs [2].

Next, we extended VBPCA so that it can extract features. In order to do this, *C*_*B*_ was assumed to have *i*,(*i*=1,…,*N*) dependence, and should be denoted as ${C_{b}^{i}}$. Thus, *P*(*B*) should also have *i* dependence as:

$$P(B_{iq}) \propto \exp \left\{- \frac{(B_{iq})^{2}}{2C_{B}^{iq}} \right\}. $$

Furthermore, it is required that *Σ*_*B*_ has *i* dependence, and should also be denoted as ${\Sigma _{B}^{i}}$. For example, in the above equations, *N* *C*_*B*_ and *N* *Σ*_*B*_ are replaced with $\sum _{i} {C_{B}^{i}}$ and $\sum _{i} {\Sigma _{B}^{i}}$, respectively.

Then $ \frac {N}{2} \log \frac {|C_{B}|}{|\Sigma _{B}|}$ in *F* is replaced with $ \frac {1}{2} \sum _{i} \log \frac {|{C_{B}^{i}}|}{|{\Sigma ^{i}_{B}}|}$. $\text {tr} \left \{ C_{B}^{-1} \left (\hat {B}^{T}\hat {B} \right) \right \}$ in *F* is replaced with $\sum _{i}\hat {\boldmath {\text {\textit {B}}}_{i}}^{T} \left ({C_{B}^{i}} \right)^{-1} \hat {\boldmath {\text {\textit {B}}}_{i}}$, where $\hat {\boldmath {\text {\textit {B}}}}_{i}$ is the *i*th row vector of $\hat {B}$. $C_{B}^{-1} N. \Sigma _{B}$ and *N* *Σ*_*B*_ in *F* are replaced with $\sum _{i} \left ({C_{B}^{i}} \right)^{-1} {\Sigma _{B}^{i}} $ and $\sum _{i} {\Sigma _{B}^{i}}$, respectively. As a result:

$${} {\small{\begin{aligned} F &= \frac{||V||_{\text{Fro}}^{2}}{2{\sigma_{E}^{2}}} + \frac{NM}{2} \log {\sigma_{E}^{2}} + \frac{M}{2} \log \frac{|C_{A}|}{|\Sigma_{A}|} + \frac{1}{2} \sum_{i} \log \frac{|{C_{B}^{i}}|}{|{\Sigma^{i}_{B}}|} \\ & + \frac{1}{2} \sum_{i} \hat{\boldmath{\text{B}}_{i}}^{T} \left ({C_{B}^{i}} \right)^{-1} \hat{\boldmath{\text{B}}_{i}} \\ & + \frac{1}{2} \text{tr} \left \{ C_{A}^{-1} \left (\hat{A}^{T}\hat{A} + M \Sigma_{A} \right) + \sum_{i} \left ({C_{B}^{i}} \right)^{-1} {\Sigma_{B}^{i}} \right. \\ &+ \left.\sigma_{E}^{-2} \left (- 2 \hat{A}^{T} V^{T} \hat{B} + \left(\hat{A}^{T}\hat{A} + M \sigma_{A} \right)\left(\hat{B}^{T}\hat{B} + \sum_{i} {\Sigma_{B}^{i}} \right) \right) \right \} \\ &+ \text{const.} \end{aligned}}} $$

is obtained. Reflecting these extensions, the above iteration rules are modified as:

(1) $$\begin{array}{@{}rcl@{}} \Sigma_{A} &\leftarrow& {\sigma_{E}^{2}} \left(\hat{B}^{T} \hat{B} + \sum_{i} {\Sigma_{B}^{i}} + {\sigma_{E}^{2}} C_{A}^{-1} \right)^{-1}  \\ \hat{A} & \leftarrow & V^{T} \hat{B} \frac{\Sigma_{A}}{{\sigma_{E}^{2}}} \\ {\Sigma_{B}^{i}} & \leftarrow & {\sigma_{E}^{2}} \left (\hat{A}^{T} \hat{A} + M \Sigma_{A} + {\sigma_{E}^{2}} \left({C_{B}^{i}}\right)^{-1} \right)^{-1}, i=1,\ldots,N \\ \hat{\boldmath{\text{B}}}_{i} & \leftarrow & \boldmath{\text{V}}_{i} \hat{A} \frac{{\Sigma_{B}^{i}}}{{\sigma_{E}^{2}}}, i=1,\ldots,N\\ \tilde{C}_{A}^{q} & \leftarrow & \frac{||\boldmath{\hat{\text{A}}}_{q}||^{2}}{M}+ (\Sigma_{A})_{qq}, q=1,\ldots,Q \\ {C_{A}^{q}} & \leftarrow& \frac{\tilde{C}_{A}^{q}}{\sum_{q'} \tilde{C}_{A}^{q'}}, q=1, \ldots,Q \\ C_{B}^{iq} & \leftarrow& (B_{iq})^{2}+ \left({\Sigma_{B}^{i}}\right)_{qq}, q=1,\ldots,Q, i=1,\ldots,N  \end{array} $$

$$\begin{array}{*{20}l}{} {\sigma_{E}^{2}} & \leftarrow \frac{1}{NM} \left \{ ||V||_{\text{Fro}}^{2} - \text{tr} \left(2V^{T}\hat{B}\hat{A}^{T} \right) \right. \\ &+ \left. \text{tr} \left (\left(\hat{A}^{T} \hat{A} + M \Sigma_{A} \right) \left(\hat{B}^{T}\hat{B} + \sum_{i} {\Sigma_{B}^{i}} \right)\right)\right\} , \end{array} $$

where *V*_*i*_ is the *i*th row *V* vector, and $\boldmath {\hat {\text {\textit {A}}}}_{q}$ is the *q*th columnar vector of matrix $\hat {A}$. Because of extension, divergence was suppressed by introducing *C*_*A*_ normalization instead of using constant values. Therefore, ${C_{b}^{i}}$ expresses *i*th feature importance, and *C*_*A*_ (instead of the former *C*_*B*_) represents importance of the *q*th PC, and must be non-constant. The order of iterations in eq. (1) is arbitrary since each iteration is independent of one another.

In the present study, the above iterations began by substituting pre-computed PCs (using the prcomp function in R[20]) in *A* and *B*, to compensate for slow convergence. After a suitable number of iterations, parameters with the smallest *F* values were used for further analysis. Convergence was judged if extracted features change after more than 100 iterations.

R code for this algorithm is available in Additional file 2.

### Statistical analysis of CPCAFE and VBPCAFE applied to simulated data

For VBPCAFE, the top-ranked features with larger $C_{B}^{i1}$ values were extracted after convergence, judged by changes in extracted features after more than 100 iterations. For CPCAFE [22-29], expression data ({*x*_*ij*_}) was embedded into low dimensional space using PCA. After selecting the PC for FE, outliers along the PC were extracted i. e., the top 10 features with larger absolute projections to the selected PC.

### Evaluation of FE performance

In order to evaluate FE performance in a simulated categorical multiclass data set, two measures were used for discrimination of binary classes with unequal member numbers. In this computation, true positive (TP) represents the number of features with distinct expression between classes (*i*≤*N* ^′^), which are identified by the specified feature. Conversely, true negative (TN) is the number of features with no distinct expression between classes (*i*>*N* ^′^), and are not identified by the specified feature. False positive (FP) is the number of features with no distinct expression between classes (*i*>*N* ^′^), but are identified by the specified feature. False negative (FN) is the number of features with distinct expression between classes (*i*≤*N* ^′^), but are not identified by the specified feature. Accordingly, the Matthews correlation coefficient is defined as:

$$\frac{TP\cdot TN - FP \cdot FN}{\sqrt{(FP+FN)(FP+TP)(TN+TP)(TN+FN)}}, $$

while the *F* measure is:

$$\frac{2 TP}{(TP+FP)+(TP+FN)}, $$

where *T* *P*+*F* *P* corresponds to the number of features identified by specific FE, and *T* *P*+*F* *N* represents the number of features with distinct expression between classes (*i*≤*N* ^′^), thus representing the harmonic mean of sensitivity $\left (\frac {TP}{TP+FN}\right)$ and precision $\left (\frac {TP}{TP+FP}\right)$.

### Translational application to PTSD associated heart disease

**Figure 16:**
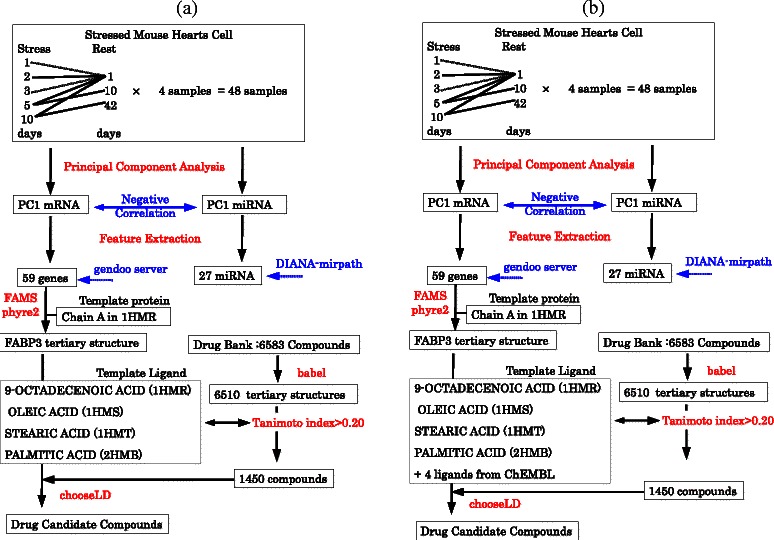
Overall study work flow. The methods used for data processing and evaluation are indicated in red and blue, respectively. Dotted lines in sample descriptions (i.e., 1 or 2 day(s) stress vs 1 day rest) indicate missing control samples (see Methods for more detail).

The overall work flow is illustrated in Figure 16.

#### mRNA and miRNA expression

mRNA and miRNA expression data were obtained from the Gene Expression Omnibus (GEO) using the GEO ID: GSE52875 [8]. Expression was observed in stressed mouse hearts. The length of time subservient mice were exposed to aggressor mice varied, with variable rest times after exposure. Exposure and rest times were 1, 2, 3, or 10 days of exposure and 1 day of rest, 5 days of exposure and 1 or 10 days of rest, and 10 days of exposure and 42 days of rest (in total, seven distinct treatment conditions). Controls were housed separately from the aggressor mice in personal cages, and prepared for all treatment conditions except for 1 or 3 days of exposure and 1 day of rest. Thus, there were only five control conditions. For each condition there were four biological replicates and therefore 48 samples in total were available.

mRNA expression was included in the subseries GSE52866 and GSE52871. GSE52866_RAW.tar and GSE52871_RAW.tar (provided as Supplementary Data in GEO) were downloaded, and the gProcessedSignal in each of 48 GSM files used for analysis. miRNA expression was included in the subseries GSE52869 and GSE52872. From GSE52872, eight raw Exiqon files (provided as Supplementary Data in GEO) were downloaded, and gProcessedSignal and rProcessedSignal used as miRNA profiles for further analysis. From GSE52869, 16 files (provided as Supplementary Data in GEO) were downloaded, and “Spot Mean Intensity Cyanine3” and “Spot Mean Intensity Cyanine5H” used as miRNA profiles for further analysis. The conditions attributed to each sample were provided by the file names. mRNA and miRNA expression were not normalized, apart from for CPCAFE, where mRNA expression was normalized to have zero mean and a standard deviation of 1.

#### KEGG pathway analysis of miRNAs using DIANA-mirpath

For CPCAFE, the 27 identified miRNAs were uploaded to the DIANA-mirpath server [11]. Although DIANA-mirpath requires mature miRNA names used in miRBase (rel. 18), the mature miRNA names in GEO ID: GSE52875 were used in the previous release, therefore the uploaded mature miRNAs are not exactly the same as the 27 identified miRNAs. Target gene prediction was performed using Tarbase [30], a database using experimentally validated targets that are more biologically plausible. Combined target genes sets were used for KEGG pathway enrichment analysis. False discovery rate corrected *P*-values were used to screen KEGG pathways. Direct weblinks to DIANA-mirpath results are provided in Additional file 10. The same analyses were performed for categorical regression-based FE and BAHSIC, excluding the number of uploaded miRNAs.

#### Disease association analysis of genes using the Gendoo server

The Gendoo server [31], a literature-based disease association database, was used to identify associations between selected mRNAs and disease. As mRNAs were identified using RefSeq mRNA IDs, these were interpreted to gene symbols. Obtained gene symbols were uploaded to the Gendoo server with mouse being the specified target species. Human was also tested for categorical regression-based FE and BAHSIC, to compensate for the small number of hits.

### KEGG pathway analysis of mRNAs by DAVID

Employed 24 genes were mRNAs that have non zero values in the column named as “target” of the “CPCA based” sheet in Additional file 4. Refseq IDs for these 24 genes were identified and were uploaded to DAVID [13] server using default setting. Then KEGG pathways identified were extracted.

#### Tertiary protein structure prediction

Tertiary protein structures were predicted using two prediction servers, Protein Homology/analogY Recognition Engine V2.0 (phyre2) [32] and full automatic modeling systems (FAMS) [33]. Among the 26 genes investigated, 14 genes were included in the PDB [34]. Tertiary protein structures of the remaining genes were predicted by either phyre2 or FAMS. The complete list of template proteins and amino acid sequences of investigated genes in fasta format is available in Additional file 11.

#### *In silico* drug discovery for FABP3

ChooseLD [35] was used for FABP3 drug discovery. Using the PDB structure of chain A in 1HMR as the template protein, and four ligands (9-OCTADECENOIC ACID in 1HMR, OLEIC ACID in 1HMS, STEARIC ACID in 1HMT, and PALMITIC ACID in 2HMB) as template ligands (acids not provided in 1HMR were mapped to 1HMR prior to chooseLD execution), with four additional FABP3 inhibitors from ChEMBL [36] as template ligands (see Additional file 2 for more detail), drug candidate compounds were selected from DrugBank [37]. ChooseLD was applied to 1450 compounds with a Tanimoto index >0.20 from at least one of the eight template ligands. The 1450 compounds were selected from 6510 compounds with tertiary structures computed by Babel software [38], from among the 6583 compounds listed in DrugBank [37]. The 1450 compounds were ranked based on Finger Print Alignment Scores (FPAScores) averaged over three independent runs.

### Generation of a test PTSD data set

In order to test equivalence between CPCAFE and VBPCAFE on PTSD data, a test data set was generated. In addition to the 100 probes selected by CPCAFE, an additional 100 probes were chosen from those remaining, generating a test data set with 200 features. VBPCAFE was applied to the generated data set, and $C_{B}^{i1}$s values calculated. The top-ranked 200 features with largest $C_{B}^{i1}$s values were selected as extracted features. FE was performed over 100 independent ensembles, and the frequency (number of times each feature was selected) counted. Frequency number = 100, indicates that the feature was always selected.

### FE cross-validation

In order to test FE stability on the PTSD data set, a 4-fold cross-validation was performed, with each experiment repeated four times. For each experimental setup, three out of four replicates were randomly selected, generating a data set with 36 samples. Since the total possible number of independent samplings was 4^12^≃2×10^7^, it is impossible to test all samplings. Therefore, 100 samplings were tested, which was large enough to demonstrate superior stability of CPCAFE compared with the two conventional supervised methods of categorical regression-based FE and BAHSIC.

### Significance of correlation coefficients

Correlation coefficient significance was investigated by transforming the correlation coefficient (*r*) to the statistical test variable (*t*):

$$t = \frac{r \sqrt{M-2}}{\sqrt{1-r^{2}}}, $$

obeying the *t* distribution of *M*−2 degrees of freedom. As there were 48 samples in our study, *M*= 48. Computed *P*-values were adjusted by BH criterion [19]. Correlation coefficients with adjusted *P*-values <0.05 were regarded as significant.

## Additional files

Additional file 1
**Confusion tables for simulated data.** Performance of FE methods applied to a simulated data set and expressed using confusion tables.

Additional file 2
**Supplementary text.** Supplementary discussion not included in the main text.

Additional file 3
**Complete DIANA-mirpath results.** Complete list of the KEGG pathway analysis reported by DIANA-mirpath for CPCAFE, categorical regression-based FE, and BAHSIC (xlsx). A partial list for CPCAFE is included in the main text as Table 3.

Additional file 4
**Correlation coefficients between selected miRNAs and target mRNAs.** Correlation coefficients between selected miRNAs and target mRNAs, with RefSeq IDs and gene symbols provided. None are filled between miRNAs and off target genes. The column “target” indicates the number of miRNAs targeting each gene (xlsx). CPCAFE, 27 miRNAs vs 59 mRNAs; categorical regression-based FE, 77 miRNAs vs 23 mRNAs; and BAHSIC, 47 miRNAs vs 37 mRNAs.

Additional file 5
**List of associated diseases with genes and miRNAs.** List of associated diseases with genes and miRNAs targeting each gene identified by CPCAFE. Lists genes not included in Table 5.

Additional file 6
**KEGG pathway mapping diagram of identified genes.** KEGG pathway mapping diagram of genes identified by CPCAFE (red characters). Genes listed as targets of drug candidate compounds in Table 7 are also indicated (blue characters).

Additional file 7
**Summary of predicted or PDB protein tertiary structures.** Summary of predicted or PDB protein tertiary structures of 26 genes with reported involvement in heart disease (see Table 5 or Additional file 4).

Additional file 8
**Full list of drug candidate compounds ranked by FPAScores.**

Additional file 9
**Stability analysis.** Stability analysis of CPCAFE, categorical regression-based FE, and BAHSIC. Frequency represents the number of times each probe was selected among 100 independent ensembles. Number of probes represents the number of probes selected by the corresponding frequency. Numbers in bold represent the number of mRNAs/miRNAs selected 100%. Note that no mRNA was selected 100% by BAHSIC.

Additional file 10
**DIANA-mirpath link.** Weblink retrieving the DIANA-mirpath results of this study.

Additional file 11
**Amino acid sequences uploaded to FAMS/phyre2.** Amino acid sequences in fasta format that were uploaded to FAMS/phyre2 to determine tertiary protein structures.

## Abbreviations

BAHSIC: Backward elimination using Hilbert-Schmidt norm of the cross covariance operator
BH: Benjamini and Hochberg
CDK2: Cyclin-dependent kinase 2
CPCA: Conventional PCA
CHD: Coronary heart disease
CPCAFE: Conventional PCA-based unsupervised FE
DAVID: Database for annotation, visualization and integrated discovery
DIANA: DNA intelligent Analysis
FABP3: Fatty acid binding protein 3
FAMS: Full automatic modeling systems
FE: Feature extraction
FP: False positive
FPAScores: Finger print alignment scores
FN: False negative
GEO: Gene expression omnibus
HSP90: Heat shock protein 90
KEGG: Kyoto encyclopedia of genes and genomes
miRNA: microRNA
PC: Principal component
PCA: principal component analysis
PDB: Protein data bank
phyre2: Protein homology/analogy recognition engine V2.0
PPAR: Peroxisome proliferator-activated receptor
PTSD: Posttraumatic stress disorder
TP: True positive
TN: True nagative
VB: Variational Bayes
VBPCAFE: VBPCA-based unsupervised FE

## References

[1] Tibshirani R (1996). Regression shrinkage and selection via the lasso. J R Stat Soc. Ser B (Methodological).

[2] Bishop CM (1999). Variational principal components. Proceedings of International Conference on Artificial Neural Networks.

[3] Lim YJ, Teh TW. Variational bayesian approach to movie rating prediction. In: Proceedings of KDD Cup and Workshop: 2007. http://www.cs.uic.edu/~liub/KDD-cup-2007/proceedings/variational-Lim.pdf.

[4] Yehuda R (2002). Post-traumatic stress disorder. N Engl J Med..

[5] Edmondson D, Kronish IM, Shaffer JA, Falzon L, Burg MM (2013). Posttraumatic stress disorder and risk for coronary heart disease: a meta-analytic review. Am Heart J..

[6] Jordan HT, Stellman SD, Morabia A, Miller-Archie SA, Alper H, Laskaris Z (2013). Cardiovascular disease hospitalizations in relation to exposure to the September 11, 2001 World Trade Center disaster and posttraumatic stress disorder. J Am Heart Assoc..

[7] Vaccarino V, Goldberg J, Rooks C, Shah AJ, Veledar E, Faber TL (2013). Post-traumatic stress disorder and incidence of coronary heart disease: a twin study. J Am Coll Cardiol..

[8] Cho JH, Lee I, Hammamieh R, Wang K, Baxter D, Scherler K (2014). Molecular evidence of stress-induced acute heart injury in a mouse model simulating posttraumatic stress disorder. Proc Natl Acad Sci USA..

[9] Kanehisa M, Goto S, Sato Y, Kawashima M, Furumichi M, Tanabe M (2014). Data, information, knowledge and principle: back to metabolism in KEGG. Nucleic Acids Res..

[10] Song L, Smola A, Gretton A, Bedo J, Borgwardt K (2012). Feature selection via dependence maximization. J Machine Learning Res..

[11] Vlachos IS, Kostoulas N, Vergoulis T, Georgakilas G, Reczko M, Maragkakis M (2012). DIANA miRPath v.2.0: investigating the combinatorial effect of microRNAs in pathways. Nucleic Acids Res..

[12] Lee HC, Chen CY, Au LC (2011). Systemic comparison of repression activity for miRNA and siRNA associated with different types of target sequences. Biochem Biophys Res Commun..

[13] Huang DAW, Sherman BT, Lempicki RA (2009). Systematic and integrative analysis of large gene lists using DAVID bioinformatics resources. Nat Protoc..

[14] Rosca MG, Vazquez EJ, Kerner J, Parland W, Chandler MP, Stanley W (2008). Cardiac mitochondria in heart failure: decrease in respirasomes and oxidative phosphorylation. Cardiovasc Res..

[15] Zhang H, Zhou L, Yang R, Sheng Y, Sun W, Kong X (2006). Identification of differentially expressed genes in human heart with ventricular septal defect using suppression subtractive hybridization. Biochem Biophys Res Commun..

[16] Kapustian LL, Vigontina OA, Rozhko OT, Ryabenko DV, Michowski W, Lesniak W (2013). Hsp90 and its co-chaperone, Sgt1, as autoantigens in dilated cardiomyopathy. Heart Vessels.

[17] Finck BN (2007). The PPAR regulatory system in cardiac physiology and disease. Cardiovasc Res..

[18] Liao HS, Kang PM, Nagashima H, Yamasaki N, Usheva A, Ding B (2001). Cardiac-specific overexpression of cyclin-dependent kinase 2 increases smaller mononuclear cardiomyocytes. Circ Res..

[19] Benjamini Y, Hochberg Y (1995). Controlling the false discovery rate: a practical and powerful approach to multiple testing. J Royal Stat Soc..

[20] R Core Team (2014). R: A Language and Environment for Statistical Computing.

[21] Tipping ME, Bishop CM (1999). Probabilistic principal component analysis. J R Stat Soc: Ser B (Stat Methodology).

[22] Kinoshita R, Iwadate M, Umeyama H, Taguchi YH (2014). Genes associated with genotype-specific DNA methylation in squamous cell carcinoma as candidate drug targets. BMC Syst Biol..

[23] Taguchi YH, Murakami Y (2013). Principal component analysis based feature extraction approach to identify circulating microRNA biomarkers. PLoS ONE.

[24] Ishida S, Umeyama H, Iwadate M, Taguchi YH (2014). Bioinformatic Screening of Autoimmune Disease Genes and Protein Structure Prediction with FAMS for Drug Discovery. Protein Pept Lett..

[25] Murakami Y, Toyoda H, Tanahashi T, Tanaka J, Kumada T, Yoshioka Y (2012). Comprehensive miRNA expression analysis in peripheral blood can diagnose liver disease. PLoS ONE.

[26] Taguchi Y-H, Okamoto A, Shibuya T, Kashima H, Sese J, Ahmad S (2012). Principal component analysis for bacterial proteomic analysis. Pattern Recognition in Bioinformatics, Lecture Notes in Computer Science.

[27] Umeyama H, Iwadate M, Taguchi Y-H (2014). TINAGL1 and B3GALNT1 are potential therapy target genes to suppress metastasis in non-small cell lung cancer. BMC Genomics..

[28] Taguchi Y-H, Huang D-S, Han K, Gromiha M (2014). Integrative analysis of gene expression and promoter methylation during reprogramming of a non-small-cell lung cancer cell line using principal component analysis-based unsupervised feature extraction. Intelligent Computing in Bioinformatics. Lecture Notes in Computer Science.

[29] Taguchi Y-H, Murakami Y (2014). Universal disease biomarker: Can a fixed set of blood micrornas diagnose multiple diseases?. BMC Reserch Notes..

[30] Vergoulis T, Vlachos IS, Alexiou P, Georgakilas G, Maragkakis M, Reczko M (2012). TarBase 6.0: capturing the exponential growth of miRNA targets with experimental support. Nucleic Acids Res..

[31] Nakazato T, Bono H, Matsuda H, Takagi T (2009). Gendoo: functional profiling of gene and disease features using MeSH vocabulary. Nucleic Acids Res..

[32] Kelley LA, Sternberg MJ (2009). Protein structure prediction on the Web: a case study using the Phyre server. Nat Protoc..

[33] Umeyama H, Iwadate M (2004). FAMS and FAMSBASE for protein structure. Curr Protoc Bioinf..

[34] Berman HM, Westbrook J, Feng Z, Gilliland G, Bhat TN, Weissig H (2000). The Protein Data Bank. Nucleic Acids Res..

[35] Takaya D, Takeda-Shitaka M, Terashi G, Kanou K, Iwadate M, Umeyama H (2008). Bioinformatics based Ligand-Docking and in-silico screening. Chem Pharm Bull..

[36] Gaulton A, Bellis LJ, Bento AP, Chambers J, Davies M, Hersey A (2012). ChEMBL: a large-scale bioactivity database for drug discovery. Nucleic Acids Res..

[37] Law V, Knox C, Djoumbou Y, Jewison T, Guo AC, Liu Y (2014). DrugBank 4.0: shedding new light on drug metabolism. Nucleic Acids Res..

[38] O’Boyle NM, Banck M, James CA, Morley C, Vandermeersch T, Hutchison GR (2011). Open Babel: An open chemical toolbox. J Cheminform..

[39] Wang X, Zhu H, Zhang X, Liu Y, Chen J, Medvedovic M (2012). Loss of the miR-144/451 cluster impairs ischaemic preconditioning-mediated cardioprotection by targeting Rac-1. Cardiovasc Res..

[40] Goren Y, Kushnir M, Zafrir B, Tabak S, Lewis BS, Amir O (2012). Serum levels of microRNAs in patients with heart failure. Eur J Heart Fail..

[41] Matkovich SJ, Wang W, Tu Y, Eschenbacher WH, Dorn LE, Condorelli G (2010). MicroRNA-133a protects against myocardial fibrosis and modulates electrical repolarization without affecting hypertrophy in pressure-overloaded adult hearts. Circ Res..

[42] Vacchi-Suzzi C, Bauer Y, Berridge BR, Bongiovanni S, Gerrish K, Hamadeh HK (2012). Perturbation of microRNAs in rat heart during chronic doxorubicin treatment. PLoS ONE.

[43] Qiang L, Hong L, Ningfu W, Huaihong C, Jing W (2013). Expression of miR-126 and miR-508-5p in endothelial progenitor cells is associated with the prognosis of chronic heart failure patients. Int J Cardiol..

[44] Duisters RF, Tijsen AJ, Schroen B, Leenders JJ, Lentink V, van der Made I (2009). miR-133 and miR-30 regulate connective tissue growth factor: implications for a role of microRNAs in myocardial matrix remodeling. Circ Res..

[45] Sutherland LB, Thatcher JE, DiMaio JM, Naseem RH, Marshall WS, van Rooij E (2008). Dysregulation of microRNAs after myocardial infarction reveals a role of miR-29 in cardiac fibrosis. Proc Natl Acad Sci USA..

[46] Rangrez AY, Massy ZA, Metzinger-Le Meuth V, Metzinger L (2011). miR-143 and miR-145: molecular keys to switch the phenotype of vascular smooth muscle cells. Circ Cardiovasc Genet..

[47] Wang J, Huang W, Xu R, Nie Y, Cao X, Meng J (2012). MicroRNA-24 regulates cardiac fibrosis after myocardial infarction. J Cell Mol Med..

[48] van Rooij E, Sutherland LB, Liu N, Williams AH, McAnally J, Gerard RD (2006). A signature pattern of stress-responsive microRNAs that can evoke cardiac hypertrophy and heart failure. Proc Natl Acad Sci USA..

[49] Ganesan J, Ramanujam D, Sassi Y, Ahles A, Jentzsch C, Werfel S (2013). MiR-378 controls cardiac hypertrophy by combined repression of mitogen-activated protein kinase pathway factors. Circulation.

[50] Wong SS, Ritner C, Ramachandran S, Aurigui J, Pitt C, Chandra P (2012). miR-125b promotes early germ layer specification through Lin28/let-7d and preferential differentiation of mesoderm in human embryonic stem cells. PLoS ONE.

[51] Tijsen AJ, Creemers EE, Moerland PD, de Windt LJ, van der Wal AC, Kok WE (2010). MiR423-5p as a circulating biomarker for heart failure. Circ Res..

[52] Bao MH, Feng X, Zhang YW, Lou XY, Cheng Y, Zhou HH (2013). Let-7 in cardiovascular diseases, heart development and cardiovascular differentiation from stem cells. Int J Mol Sci..

[53] Spinetti G, Fortunato O, Caporali A, Shantikumar S, Marchetti M, Meloni M (2013). MicroRNA-15a and microRNA-16 impair human circulating proangiogenic cell functions and are increased in the proangiogenic cells and serum of patients with critical limb ischemia. Circ Res..

[54] Zhang ZH, Li J, Liu BR, Luo CF, Dong Q, Zhao LN (2013). MicroRNA-26 was decreased in rat cardiac hypertrophy model and may be a promising therapeutic target. J Cardiovasc Pharmacol..

[55] Crippa S, Cassano M, Messina G, Galli D, Galvez BG, Curk T (2011). miR669a and miR669q prevent skeletal muscle differentiation in postnatal cardiac progenitors. J Cell Biol..

[56] Miwa K, Lee JK, Takagishi Y, Opthof T, Fu X, Hirabayashi M (2013). Axon guidance of sympathetic neurons to cardiomyocytes by glial cell line-derived neurotrophic factor (GDNF). PLoS ONE.

[57] Chan AO, Jim MH, Lam KF, Morris JS, Siu DC, Tong T (2007). Prevalence of colorectal neoplasm among patients with newly diagnosed coronary artery disease. JAMA.

[58] Kerkela R, Grazette L, Yacobi R, Iliescu C, Patten R, Beahm C (2006). Cardiotoxicity of the cancer therapeutic agent imatinib mesylate. Nat Med..

[59] Patwary MS, Haque KMHSS, Shoaib N, Salehin KS, Hosan ATMI (2012). Cardiac Involvement of Hepatitis B and C Virus Infection. Univ Heart J..

[60] van Haelst PL, Schot B, Hoendermis ES, van den Berg MP (2006). Acute myeloid leukaemia as a cause of acute ischaemic heart disease. Neth Heart J..

[61] DiMichele LA, Doherty JT, Rojas M, Beggs HE, Reichardt LF, Mack CP (2006). Myocyte-restricted focal adhesion kinase deletion attenuates pressure overload-induced hypertrophy. Circ Res..

[62] Muslin AJ (2008). MAPK signalling in cardiovascular health and disease: molecular mechanisms and therapeutic targets. Clin Sci..

[63] Ward KK, Shah NR, Saenz CC, McHale MT, Alvarez EA, Plaxe SC (2012). Cardiovascular disease is the leading cause of death among endometrial cancer patients. Gynecol Oncol..

[64] Bonney KM, Engman DM (2008). Chagas heart disease pathogenesis: one mechanism or many?. Curr Mol Med..

[65] Xu Y, Li X, Liu X, Zhou M (2010). Neuregulin-1/ErbB signaling and chronic heart failure. Adv Pharmacol..

[66] Thomas JA, Gerber L, Banez LL, Moreira DM, Rittmaster RS, Andriole GL (2012). Prostate cancer risk in men with baseline history of coronary artery disease: results from the REDUCE Study. Cancer Epidemiol Biomarkers Prev..

[67] Leak D, Meghji M (1979). Toxoplasmic infection in cardiac disease. Am J Cardiol..

[68] Bujak M, Frangogiannis NG (2007). The role of TGF-beta signaling in myocardial infarction and cardiac remodeling. Cardiovasc Res..

[69] Steingart RM, Bakris GL, Chen HX, Chen MH, Force T, Ivy SP (2012). Management of cardiac toxicity in patients receiving vascular endothelial growth factor signaling pathway inhibitors. Am Heart J..

[70] Perez-Lloret S, Rey MV, Crispo J, Krewski D, Lapeyre-Mestre M, Montastruc JL (2014). Risk of heart failure following treatment with dopamine agonists in Parkinson’s disease patients. Expert Opin Drug Saf..

[71] Depression and Heart Disease. http://www.nimh.nih.gov/health/publications/depression-and-heart-disease/index.shtml.

[72] Trifilo MJ, Yajima T, Gu Y, Dalton N, Peterson KL, Race RE (2006). Prion-induced amyloid heart disease with high blood infectivity in transgenic mice. Science.

[73] Razani B, Zhang H, Schulze PC, Schilling JD, Verbsky J, Lodhi IJ (2011). Fatty acid synthase modulates homeostatic responses to myocardial stress. J Biol Chem..

[74] Severs NJ, Coppen SR, Dupont E, Yeh HI, Ko YS, Matsushita T (2004). Gap junction alterations in human cardiac disease. Cardiovasc Res..

