# Supplementary material for: Principal component analysis-based unsupervised feature extraction applied to in silico drug discovery for posttraumatic stress disorder-mediated heart disease
Source: BMC Bioinformatics. 2015 Apr 30;16:139. doi: 10.1186/s12859-015-0574-4 (PMC4448281; doi:10.1186/s12859-015-0574-4)
Supplement: Additional file 2 — Supplementary text. Supplementary discussion not included in the main text. [file 12859_2015_574_MOESM2_ESM.pdf]

Supplementary text

Contents

1 Identification of PCs to be investigated **1**
1.1 Introduction . . . 1
1.2 Significant sample dependence of PCs . . . 2
1.3 Correlation of PCs between mRNA and miRNA . . . 2
1.4 Number of outliers along specified PCs . . . 3
1.5 Conclusion . . . 4

2 More details about Figs. 6, 8, and 9 **4**
2.1 Figs. 6 and 8 . . . 4
2.2 Fig. 9 . . . 4

3 Comparison between statistical tests using P-values (Fig. 7) and logarithmic ratio (Table 4) **4**

4 Additional details of drug discovery using chooseLD **6**

5 R code for VBPCAFE **7**

6 R code for BAHSIC **9**

1 Identification of PCs to be investigated

1.1 Introduction

In this supplementary document, we discussed the identification of PCs to be investigated. PCA usually give us more than one PCs. It is critical to identify PCs to be investigated. Since we would like to perform integrated analysis, there are some requirements for PCs to be investigated.

1. PCs should be distinct between samples.
2. PCs should be correlated between mRNA and miRNA.
3. There are enough number of outliers along PCs.

The first requirement is because PC should represent some distinction between treatments. If not, outliers along the PC are not expected to exhibit

distinction between treatments. The second requirement is because this allows us to select more biologically feasible set of mRNA/miRNAs. Several mRNAs and miRNAs can exhibit apparent distinction between treatments, but they may be simple fluctuation. If they are correlated between mRNAs and miRNAs, it is less likely to be simple noise, since noise is not expected to be correlated. Finally, the third requirement is necessary since if not enough number of mRNAs/miRNAs are selected, it is less likely that selected mRNAs/miRNAs include enough number of biologically significant ones (e.g., by literature search).

1.2 Significant sample dependence of PCs

In order to check if PCs are distinct between treatments (e.g., control vs treated samples), we applied categorical regression to PCs. Categorical regression applied to PCs is

$$PC_{ij} = C_{i0} + \sum_k \delta_{k,k(j)} C_{ik}$$

where PC_{ij} is the contribution of jth sample to ith PC, $\delta_{k,k(j)}$ is clonecker's delta, $k(j)$ is the sample category that jth sample belongs to, and $\sum_k$ runs over all sample categories. This can be evaluated by lm function in R as follows,

```
LM <- lm(PC[i] ~ factor(class))
summary(LM)
```

where PC[i] is a vector consists of contribution of jth sample to the ith PC, class is class labelling of samples. P-values obtained from this command is as follows, Thus, it is plausible to consider only the first and second PCs.

	PC1	PC2	PC3
miRNA	2×10^{-4}	2×10^{-5}	0.44
mRNA	2×10^{-6}	$< 2 \times 10^{-16}$	0.05

1.3 Correlation of PCs between mRNA and miRNA

Correlation coefficients of PCs between mRNA and miRNA is as follows. Again, considering only the first and second PCs turned out to be plausible. Thus, we will never consider the third PC and any other lower ranked PCs.

	PC1	PC2	PC3
Correlation Coefficients	-0.52	-0.70	-0.12
P-values	2×10^{-4}	4×10^{-8}	0.40

1.4 Number of outliers along specified PCs

Finally, we would like to estimate how many outliers are associated with the first or second PCs. If not enough number of outliers are associated with each PC, considering the PC is useless. At a glance (Figs. 6 and 8), most outliers are associated with the first PC. If there are very few outliers not associated with the first PC but with the second PC, considering the second PC independently of the first PC will be useless. In spite of that we extracted outliers only along the first PC as described in the main text, there are very few unique outliers along the second PC if they are also potentially outliers along the second PC. For example, there are only eight unique miRNAs (mmu-miR-208a, -20b, -216a, -30a, 590-5p, -186*, -1929, and 654-5p) and 15 mRNAs associated with unique RefSeq mRNA IDs (NM_001004193 (Rhox8), NM_001012434 (Kctd14), NM_001033337 (Ttc38), NM_001038592 (Glrx2), NM_001082476 (Ndor1), NM_007831 (Dcc), NM_007922 (Elk1), NM_008103 (Gcm1), NM_008222 (Hccs), NM_008725 (Nppa), NM_009608 (Actc1), NM_011082 (Pigr), NM_011387 (Slc10a1), NM_019494 (Cxcl11), and NM_198107 (Med16)). Besides small number of selected mRNAs/miRNAs, gendoo (see main text) could report only six genes associated with heart diseases (generally, small number of weak associations, see below).

Gene	Heart diseases	P-values
Glrx2	Atherosclerosis	0.0005
Elk1	Atherosclerosis	0.04
	Arteriosclerosis	0.002
Gcm1	Heart Defects, Congenital	0.006
Nppa	Heart Defects, Congenital	2.5×10^{-55}
	Cardiomegaly	1.1×10^{-28}
	Cardiomyopathies	7.5×10^{-17}
Actc1	Heart Defects, Congenital	2.5×10^{-38}
	Cardiomyopathy, Dilated	6.6×10^{-9}
	Heart Septal Defects	5.1×10^{-7}
Cxcl11	Vascular Diseases	0.01

Please note that these excluded mRNAs were also identified by two FEs employed to demonstrate superiority of CPCAFE. Thus, these mRNAs can also be secondary candidates for more detailed biological investigations.

1.5 Conclusion

Considering these above observations, we decided to concentrate to the first PC.

2 More details about Figs. 6, 8, and 9

2.1 Figs. 6 and 8

In Figs. 6 and 8, miRNAs and mRNAs satisfying the following conditions are extracted;

$$\mathrm{PC1}_i^k > D$$

and

$$\mid \mathrm{PC2}_i^k \mid < D$$

where $D = 5000$ and 20 for $k =$ miRNA and mRNA, respectively and $\mathrm{PC2}_i^k$ is the second principal component score of ith mRNA and miRNA. Then top ranked 100 miRNAs or mRNAs having larger $\mathrm{PC1}_i^k$ were extracted.

2.2 Fig. 9

Suppose x_{ij}^{mRNA} and x_{ij}^{miRNA} are the mRNA and miRNA expression of ith mRNA and miRNA of jth sample ($j = 1, ..., 48$). After applying principal component analysis, the first principal component scores of ith mRNA and miRNA are obtained as

$$\mathrm{PC1}_i^k = \sum_{j=1}^{48} C_j^k x_{ij}^k$$

where $k =$ mRNA or miRNA and C_j^ks are coefficeints. Fig. 9(a) is the scatter plot between C_j^k where $k =$ mRNA for horizontal axis and $k =$ miRNA for vertical axis. In Fig. 9(b), four C_j^ks corrsponding to four biological replicates under the same experimental condiction out of 12 contisons are avaraged and plotted.

3 Comparison between statistical tests using P-values (Fig. 7) and logarithmic ratio (Table 4)

In order to compare between statistical tests using P-values employed in this study (Fig. 7) and logarithmic ratio (Table 4), we have demonstrated its

performace using simulated data set. This date set consists of $2N$ samples with 20 features. $2N$ samples consist of two classes each of which includes N samples. The first 10 features have equal to or smaller expression in the first class compared with in the second class but the last 10 features do not, i.e.,

$$x_{ij} = \begin{cases} \mathcal{N}(\mu, 1) & 1 \leq i \leq 10, \quad N < j \leq 2N \\ \mathcal{N}(1, 1) & 11 \leq i \leq 20, \quad N < j \leq 2N \\ \mathcal{N}(1, 1) & 1 \leq i \leq 20, \quad 1 \leq j \leq N \end{cases}$$

where x_{ij} stands for expression of ith feature of jth sample and $\mathcal{N}(\mu, \sigma)$ is the normal distribution with $\mu(\geq 1)$ mean and σ standard deviation. The task is to test if we can distinguish between the first 10 features ($1 \leq i \leq 10$) with distinct expression between two classes and the last 10 features ($11 \leq i \leq 20$) without distinct expression between two classes. In order to do this, we attributed two statistical values to each feature. The first one (used in Table 4) is logarithmic ratio of mean expression between two classes, i.e.,

$$\log \frac{\sum_{j=1}^{N} x_{ij}}{\sum_{j=N+1}^{2N} x_{ij}}$$

The second one (used in Fig. 7) is logarithmic P-values computed by t test with the null hypothesis that mean expression is equal between two classes and the alternative hypothesis that the mean expression is smaller in the first class ($1 \leq j \leq N$) than the second class ($N < j \leq 2N$). Please note that both statistical values are expected to be smaller in average in the first 10 features ($1 \leq i \leq 10$) than in the last 10 features ($11 \leq i \leq 20$).

Then, P values that represent the significance of difference between the first 10 and the last 10 features are computed using one of two statistical values (logarithmic ratio or logarithmic P-values) with t test with the null hypothesis that mean statistical values are equal between the first 10 features and the second 10 features and the alternative hypothesis that the mean statistical values are smaller in the first 10 features ($1 \leq i \leq 10$) than in the last 10 features ($11 \leq i \leq 20$).

The figure shows the μ value dependence of averaged P-values (over 100 trials) that represent the significance of difference between the first 10 and the last 10 features for $N = 10$ (smaller number of samples) and $N = 100$ (larger number of samples). Since horizontal broken lines represent $P = 0.05$, P-values below these lines correspond to the successful discriminations between the first 10 features and the last 10 features.

N=10 N=100

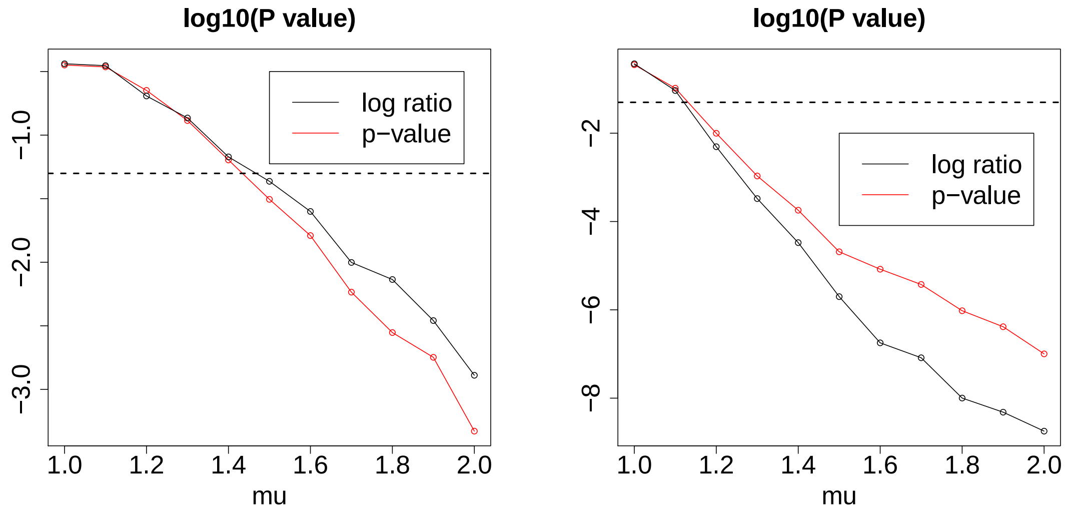

It is clear that logarithmic P-values are more sensitive (smaller) than logarithmic ratio for smaller samples ($N = 10$) when the discrimination is successful (i.e. $P < 0.05$). Since there are only four samples in each class for PTSD experiments, it is possibly the reason why we had to employ logarithmic P-values instead of logarithmic ratio for miRNAs (Fig. 7). On the other hand, the reason why logarithmic ratio was valid for mRNAs (Table 4) was possibly because the difference between treatment and control was big enough.

4 Additional details of drug discovery using chooseLD

Since template ligands consist of only acids (i.e., 9-OCTADECENOIC ACID, OLEIC ACID, STEARIC ACID, PALMITIC ACID), it was not expected for compounds other than acid to have higher FPAScores. Thus, we decided to add more compounds to template ligands.

In order to find additional template ligands, we sought compounds that bind to FABP3 using ChEMBL. In ChEMBL, FABP3 was identified as CHEMBL3344. For CHEMBL3344, there are 29 compounds with Ki values listed. Among those 29 compounds, four compounds with higher affinity to FABP3,

CHEMBL247298 (Ki = 4nM)	CID44441441
CHEMBL116533 (Ki = 32nM)	CID10001781
CEEMBL394440 (Ki = 110nM)	CID44441314
CHEMBL247529 (Ki = 220nM)	CID20577264

were selected as additional template compounds candidates. In order to include these four compounds to template ligands, we inferred binding modes to FABP3 (Chain A of PDB ID: 1HMR) using chooseLD with employing four acids as template ligands.

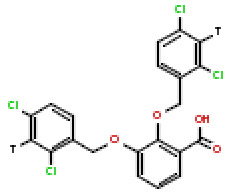
CHEMBL247298

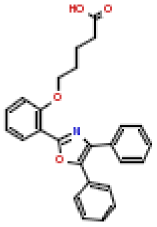
CHEMBL116533

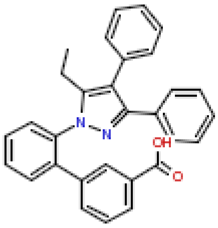
CHEMBL394440

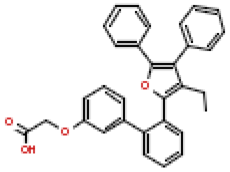
CHEMBL247529

Four compounds with higher affinity to FABP3

Then, configurations with the highest FPAScores that were attributed to compounds were employed as structures to be added as additional members of template ligands. Then chooseLD was executed toward 1450 compounds selected from DrugBank, with employing eight compounds as template ligands, i.e., 9-OCTADECENOIC ACID, OLEIC ACID, STEARIC ACID, PALMITIC ACID, CHEMBL247298, CHEMBL116533, CHEMBL394440, and CHEMBL247529.

5 R code for VBPCAFE

```
VBPCAFE <- function(V,H,N=100)
{
L <- dim(V)[1]
M <- dim(V)[2]
F_value <- function(A,B,SA,SB,CA,CB,s,L)
{
detSB <- apply(SB,3,det)
FB<-0
for (il in c(1:L))  FB <- FB+1/2*log(det(CB[,,il])/detSB[il])
DCB <- 0
for (il in c(1:L)) DCB <- DCB + B[il,] %*% solve(CB[,,il]) %*% B[il,] +
     sum(diag(SB[,,il]%*%solve(CB[,,il])))
F <- sum(V^2)/(2*s^2)+L*M/2*log(s^2)+M/2*log(det(diag(CA,H))/det(SA))+
     FB+
     1/2*sum(diag(solve(diag(CA,H))%*%(t(A)%*%A+M*SA)+
```

```
     1/s^2*(-2*t(A)%*%t(V)%*%B+
     (t(A)%*%A+M*SA)*(t(B)%*%B+apply(SB,c(1,2),sum)))))+1/2*DCB
return(F)
}
pca <- prcomp(V)
B <- pca$x[,1:H] #test data
A <- pca$rotation[,1:H] #test data
s<-1 #test data
SA <- diag(rep(1,H))
SB <- array(diag(rep(1,H)),dim=c(H,H,L))
detSB <- apply(SB,3,det)
CA <- rep(1/H,H)
CB <- rep(1,H*L)
CB <-array(apply(matrix(CB,nrow=H),2,diag),dim=c(H,H,L))
F <- F_value(A,B,SA,SB,CA,CB,s,L)
F_top <- F
A_top <- A
B_top <-B
SA_top <- SA
SB_top <- SB
CA_top <- CA
CB_top <- CB
s_top <- s
for (i in c(1:N))
{
SA<- s^2*solve(t(B)%*%B +  apply(SB,c(1,2),sum) + s^2*solve(diag(CA,H)))
A <- t(V)%*%B%*%SA/s^2
SB1 <- array(NA,dim=c(H,H,L))
for (il in c(1:L)) SB1[,,il] <- s^2*solve(t(A)%*%A + M* SA +
                                                    s^2*solve(CB[,,il]))
SB <- SB1
B1 <- NULL
for (il in c(1:L)) B1 <- rbind(B1,V[il,] %*% A %*% SB[,,il]/s^2)
B<-B1
CA <- colSums(A^2)/M+diag(SA)
CA <- CA/sum(CA)
CB1 <- array(NA,dim=c(H,H,L))
for (il in c(1:L)) CB1[,,il] <- diag(B[il,]^2+diag(SB[,,il]))
CB<-CB1
s <- ((sum(diag(t(V)%*%V)) - sum(diag(2*t(V)%*%B%*%t(A)))
       +sum(diag((t(A)%*%A+M*SA)%*%(t(B)%*%B+
```

```
        apply(SB,c(1,2),sum)))))/(L*M))^0.5
detSB <- apply(SB,3,det)
F <- F_value(A,B,SA,SB,CA,CB,s,L)
if (F<F_top)
{
A_top <- A
B_top <-B
SA_top <- SA
SB_top <- SB
CA_top <- CA
CB_top <- CB
s_top <- s
F_top <- F
}
index <- order(-CA*rowSums(apply(CB,3,diag)))
CA <- CA[index]
CB <- CB[index,index,]
A <- A[,index]
B <- B[,index]
SA <- SA[index,index]
SB <- SB[index,index,]
}
return(list(A=A_top,B=B_top,SA=SA_top,SB=SB_top,
            CA=CA_top,CB=CB_top,s=s_top,F=F_top))
}
```

6 R code for BAHSIC

```
#pre-loaded function
HSIC2 <- function(DDM,DLM,X,i){
DDMP <- outer(X[i,],X[i,],"*")
DM <- mean((DDM-DDMP)*DLM)+mean((DDM-DDMP))*mean(DLM)-
2*mean(outer(colMeans((DDM-DDMP)),colMeans(DLM),"*"))
return(DM)
}

#main function
#N: number of extracted features
#XX: expression matrix, row:features, column:samples
#class: vector that represents class labels of samples
```

```
#p: ratio of number of features to be discarded every step
#output is the vector whose the last N numbers represent
# the index of extracted features
BAHSIC <- function(XX,N,class,p=0.1)
{
DDM <- t(XX)%*%XX
DLM <- outer(class,class,"==")
H <- NULL;for(i in c(1:dim(XX)[1]))
{H <- c(H,HSIC2(DDM,DLM,XX,i))}
SS <- order(-H)[1:(length(H)*p)]
DDM <- t(XX[-SS,])%*%XX[-SS,]
while(dim(XX)[1]-length(SS)>N)
{
H <- rep(NA,dim(XX)[1]);for(i in c(1:dim(XX)[1])[-SS])
 {H[i] <- HSIC2(DDM,DLM,XX,i)}
SS <- c(SS,order(-H)[1:(sum(!is.na(H))*0.1)])
DDM <- t(XX[-SS,])%*%XX[-SS,]
}
SS <-c(SS,c(1:dim(XX)[1])[-SS])
return(SS)
}
```
