# Supplementary material for: Principal component analysis-based unsupervised feature extraction applied to in silico drug discovery for posttraumatic stress disorder-mediated heart disease
Source: BMC Bioinformatics. 2015 Apr 30;16:139. doi: 10.1186/s12859-015-0574-4 (PMC4448281; doi:10.1186/s12859-015-0574-4)
Supplement: Additional file 5 — List of associated diseases with genes and miRNAs. List of associated diseases with genes and miRNAs targeting each gene identified by CPCAFE. Lists genes not included in Table 5. [file 12859_2015_574_MOESM5_ESM.pdf]

Table 1: 15 genes targeted by at least one of selected 27 miRNAs

Gene	RefSeq mRNA	miRNA that taregt miRNA
Synrg	NM_194341	miR-451, miR-22-3p, miR-709, miR-143-3p, miR-126-5p, miR-16, miR-1983, miR-207
Uba52	NM_019883	miR-133a-3p, miR-133b-3p
Crip2	NM_024223	miR-709
Uqcrq	NM_025352	miR-709
1700047I17Rik2	NM_001100116	miR-30c-5p, miR-29a-3p, miR-133a-3p, miR-30b-5p, miR-29b-3p, miR-16,miR-30e-5p, miR-1983
Rplp0	NM_007475	miR-30c-5p, miR-30b-5p,miR-30e-5p
Hbb-bt	NM_008220	miR-143-3p
Ndufa13	NM_023312	miR-24-3p
Pebp1	NM_018858	miR-24-3p
Exosc2	NM_144886	miR-378a-5p
Pbld1	NM_026701	miR-125b-5p
2610028H24Rik	NM_029816	miR-125b-5p
Medag	NM_027519	let-7b-5p, let-7g-5p
Tpt1	NM_194341	miR-16
Uqcrh	NM_025641	miR-691

Table 2: 17 genes associated with heart diseases but not targeted by any of selected 27 miRNAs

RefSeq mRNA	Gene	Heart disease association (P-values)
NM_007450	*Slc25a4*	Cardiomyopathies (3.3×10^{-5}) ; Cardiomegaly (1.6×10^{-2})
NM_001164248	*Tpm1*	Cardiomyopathy, Hypertrophic, Familial (6.0^{-18}), Cardiomyopathy, Dilated (9.6×10^{-5}), Cardiomyopathy, Hypertrophic (5.2×10^{-3}), Heart Diseases (9.1×10^{-3})
NM_001164171	*Myh6*	Cardiomyopathy, Hypertrophic (4.9×10^{-42}), Heart Defects, Congenital (2.5×10^{-28}), Cardiomyopathy, Dilated (3.0×10^{-12}), Cardiomegaly (3.1×10^{-12})
NM_008218	*Hba-a1*	Cardiomegaly (1.7×10^{-2})
NM_013593	*Mb*	Myocardial Infarction (9.0×10^{-52}), Myocardial Ischemia (4.8×10^{-18}), Myocardial Reperfusion Injury (8.8×10^{-11}),Angina Pectoris (8.5×10^{-17})
NM_011619	*Tnnt2*	Cardiomyopathy, Hypertrophic (5.9×10^{-17}), Cardiomyopathy, Dilated (1.1×10^{-11}), Cardiomyopathy, Hypertrophic, Familial (4.0×10^{-10}), Ventricular Dysfunction, Left (3.7×10^{-7})
NM_008084	*Gapdh*	Cardiomyopathy, Dilated (1.6×10^{-3}), Heart Diseases (9.6×10^{-3}), Myocardial Infarction (3.4×10^{-2}), Heart Defects, Congenital (3.4×10^{-2})
NM_009944	*Cox7a1*	Chagas Cardiomyopathy (1.2×10^{-4}), Myocarditis (1×10^{-3})
NM_175329	*Chchd10*	Cardiovascular Diseases (3.1×10^{-3})
NM_016774	*Atp5b*	Cardiomyopathies (3.8×10^{-3})
NM_008653	*Mybpc3*	Cardiomyopathy, Hypertrophic (1.7×10^{-11}), Ventricular Dysfunction, Left (1.4×10^{-7}), Cardiomyopathy, Dilated (3.5×10^{-7}), Cardiomyopathy, Hypertrophic, Familial (5.3×10^{-7})
NM_009964	*Cryab*	Cardiomyopathies (4.0×10^{-13}), Myocardial Ischemia (7.1×10^{-8}), Myocardial Reperfusion Injury (6.4×10^{-6}), Heart Failure (3.1×10^{-5})
NM_009406	*Tnni3*	Cardiomyopathy, Dilated (7.5×10^{-12}), Cardiomyopathy, Hypertrophic, Familial (3.1×10^{-10}), Ventricular Dysfunction, Left (2.9×10^{-7}), Cardiomyopathies (1.1×10^{-6})
NM_198415	*Ckmt2*	Hypertrophy, Left Ventricular (1.4×10^{-3}), Cardiomyopathy, Dilated (2.0×10^{-3}), Myocardial Reperfusion Injury (2.9×10^{-3}), Myocardial Ischemia (3.2×10^{-3})
NM_007505	*Atp5a1*	Cardiomyopathies (7.7×10^{-3})
NM_177369	*Myh8*	Heart Defects, Congenital (2.6×10^{-3})
NM_010212	*Fhl2*	Heart Defects, Congeimesnital (7.7×10^{-5}), Cardiomyopathies (1.2×10^{-2}), Cardiomegaly (2.2×10^{-2}), Myocardial Infarction (3.1×10^{-2})

Table 3: 18 genes neither associated with heart diseases nor targeted by any of selected 27 miRNAs

RefSeq	gene
NM_001083955	*Hba-a2*
NM_001033435	*Milr1*
NM_008617	*Mdh2*
NM_024166	*Chchd2*
NM_175015	*Atp5g3*
NM_026614	*Ndufa5*
NM_027862	*Atp5h*
NM_170759	*Zfp628*
NM_010888	*Ndufs6*
NM_007751	*Cox8b*
NM_010239	*Fth1*
NM_173011	*Idh2*
NM_023374	*Sdhb*
NM_053071	*Cox6c*
NM_025983	*Atp5e*
NM_080633	*Aco2*
NM_031165	*Hspa8*
NM_020582	*Atp5j2*
