# Supplementary material for: Principal component analysis-based unsupervised feature extraction applied to in silico drug discovery for posttraumatic stress disorder-mediated heart disease
Source: BMC Bioinformatics. 2015 Apr 30;16:139. doi: 10.1186/s12859-015-0574-4 (PMC4448281; doi:10.1186/s12859-015-0574-4)
Supplement: Additional file 6 — KEGG pathway mapping diagram of identified genes. KEGG pathway mapping diagram of genes identified by CPCAFE (red characters). Genes listed as targets of drug candidate compounds in Table 7 are also indicated (blue characters). [file 12859_2015_574_MOESM6_ESM.pdf]

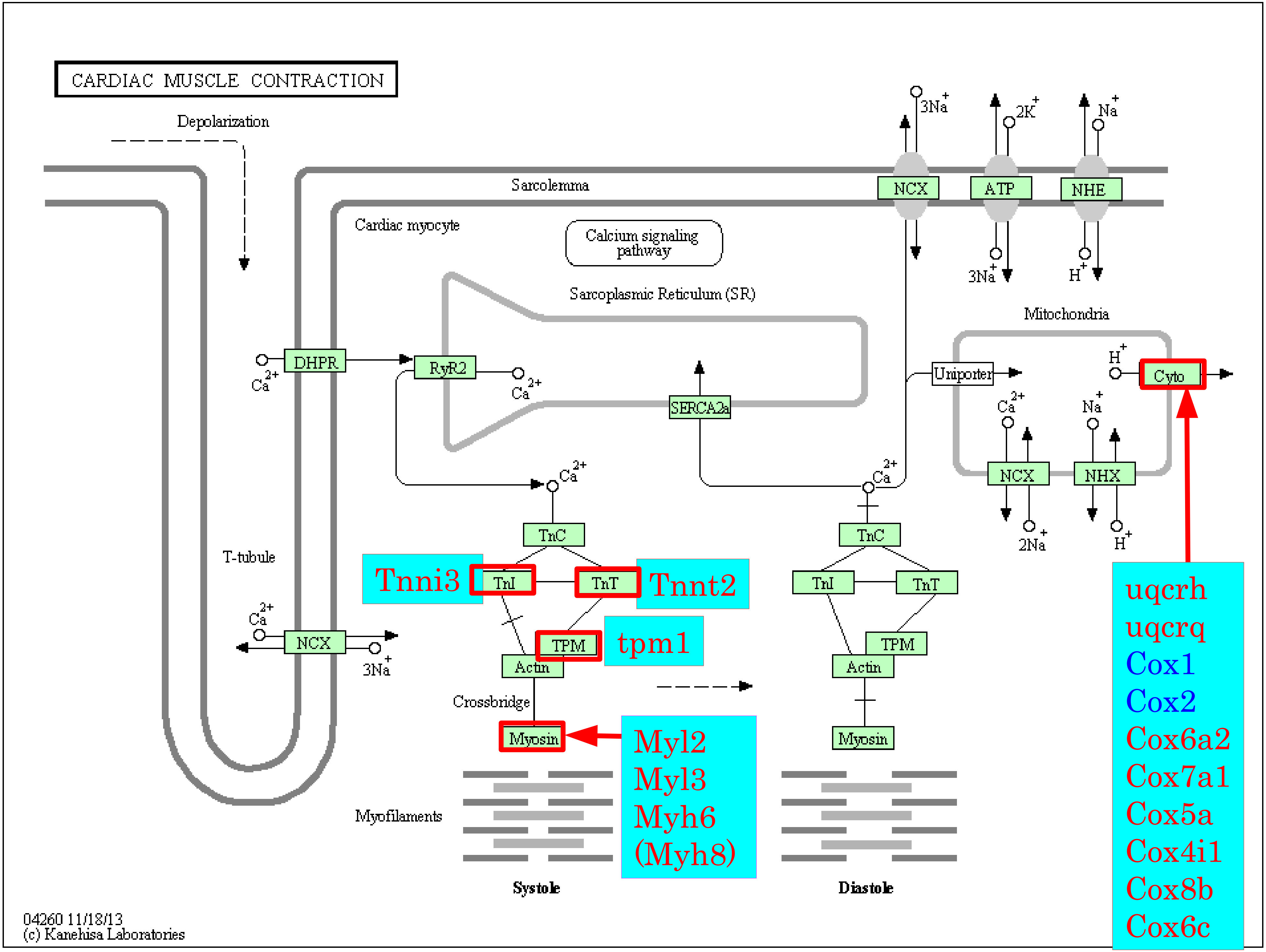
CARDIAC MUSCLE CONTRACTION
Depolarization
Sarcolemma
Cardiac myocyte
Calcium signaling pathway
Sarcoplasmic Reticulum (SR)
NCX
ATP
NHE
3Na+
2K+
Na+
H+
Mitochondria
DHPR
RyR2
SERCA2a
Ca2+
Uniporter
Cyto
NHX
2Na+
T-tubule
TnC
TnI
TnT
TPM
Actin
Myosin
Crossbridge
Myofilaments
Systole
Diastole
Tnni3
Tnnt2
tpm1
Myl2
Myl3
Myh6
(Myh8)
uqcrh
uqcrq
Cox1
Cox2
Cox6a2
Cox7a1
Cox5a
Cox4i1
Cox8b
Cox6c
04260 11/18/13
(c) Kanehisa Laboratories

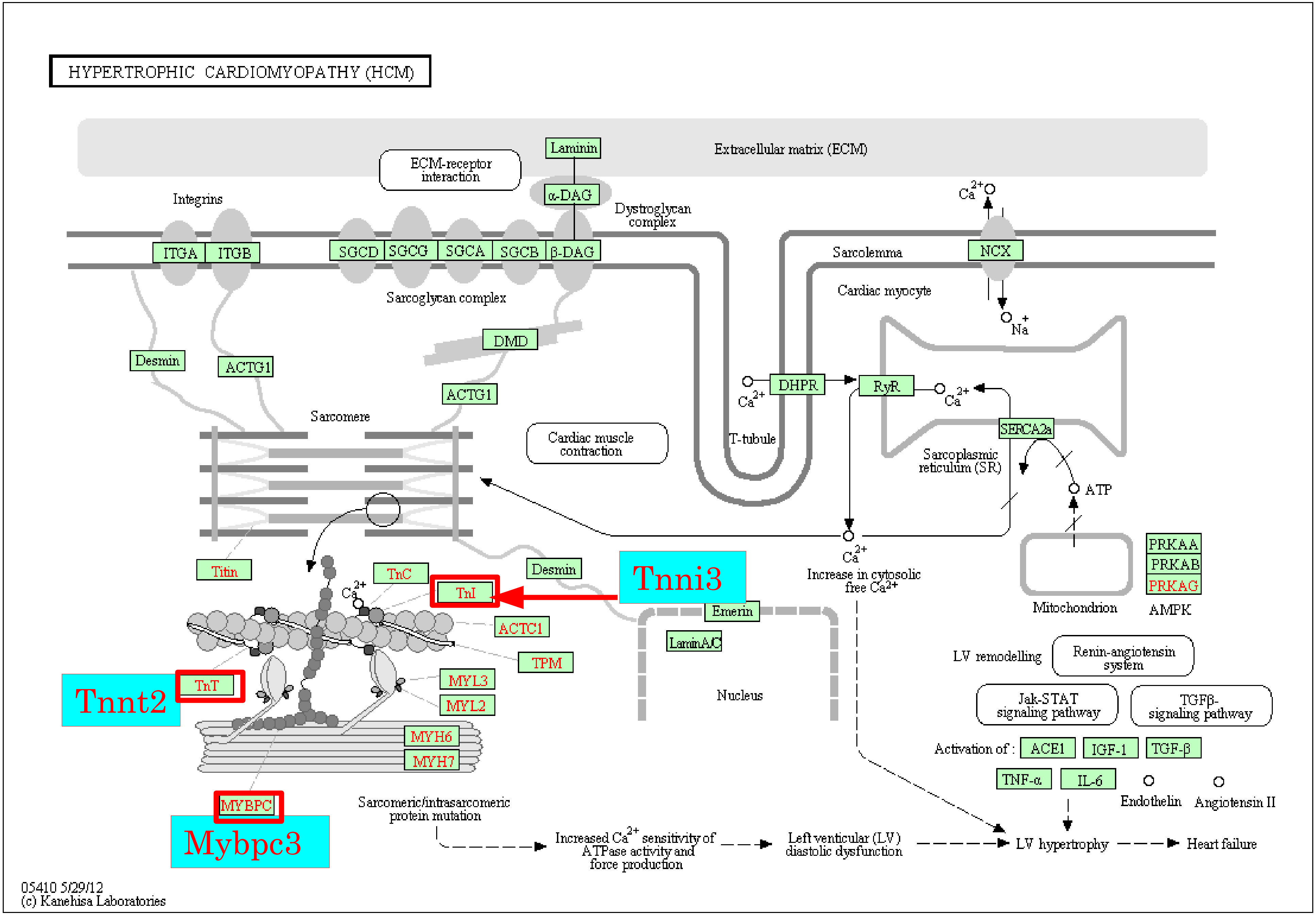

HYPERTROPHIC CARDIOMYOPATHY (HCM)
Extracellular matrix (ECM)
ECM-receptor interaction
Laminin
α-DAG
Dystroglycan complex
Integrins
ITGA
ITGB
SGCD
SGCG
SGCA
SGCB
β-DAG
Sarcoglycan complex
Sarcolemma
NCX
Cardiac myocyte
DMD
Desmin
ACTG1
ACTG1
Sarcomere
Cardiac muscle contraction
DHPR
RyR
T-tubule
SERCA2a
Sarcoplasmic reticulum (SR)
ATP
Titin
TnC
TnI
Desmin
Tnni3
Emerin
Increase in cytosolic free Ca^{2+}
PRKAA
PRKAB
PRKAG
AMPK
Mitochondrion
ACTC1
LaminA/C
TPM
Tnnt2
TnT
MYL3
MYL2
MYH6
MYH7
Nucleus
LV remodelling
Renin-angiotensin system
Jak-STAT signaling pathway
TGFβ-signaling pathway
Activation of :
ACE1
IGF-1
TGF-β
TNF-α
IL-6
Endothelin
Angiotensin II
MYBPC
Mybpc3
Sarcomeric/intrasarcomeric protein mutation
Increased Ca^{2+} sensitivity of ATPase activity and force production
Left venticular (LV) diastolic dysfunction
LV hypertrophy
Heart failure
05410 5/29/12
(c) Kanehisa Laboratories

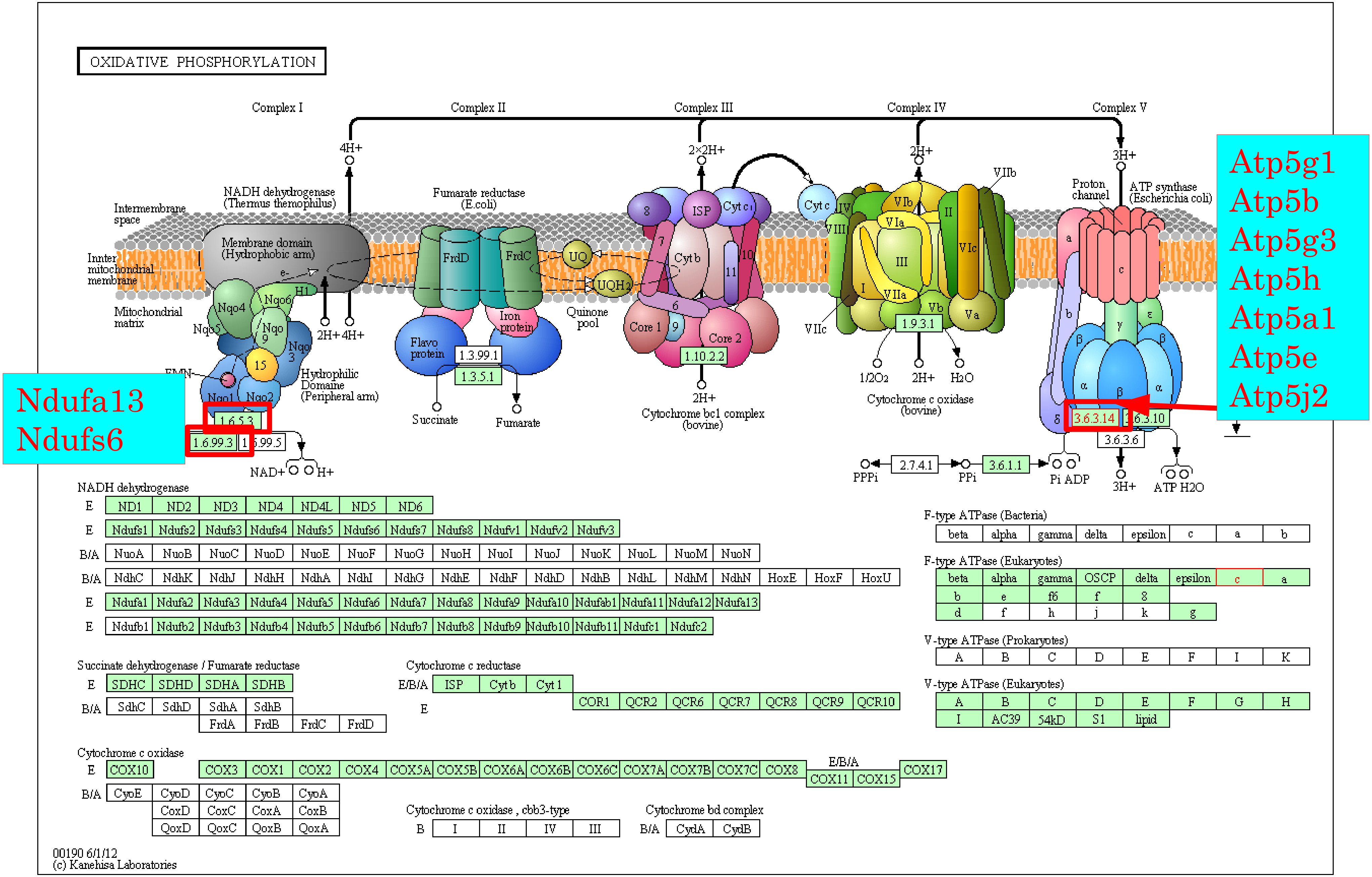

OXIDATIVE PHOSPHORYLATION
Complex I
Complex II
Complex III
Complex IV
Complex V
4H+
2×2H+
2H+
3H+
Intermembrane space
Inner mitochondrial membrane
Mitochondrial matrix
NADH dehydrogenase (Thermus themophilus)
Membrane domain (Hydrophobic arm)
Hydrophilic Domaine (Peripheral arm)
Fumarate reductase (E.coli)
FrdD
FrdC
UQ
UQH2
Quinone pool
Flavo protein
Iron protein
Succinate
Fumarate
Cytochrome bc1 complex (bovine)
Cytochrome c oxidase (bovine)
Proton channel
ATP synthase (Escherichia coli)
1.6.5.3
1.6.99.3
1.6.99.5
1.3.99.1
1.3.5.1
1.10.2.2
1.9.3.1
3.6.3.14
3.6.3.10
3.6.3.6
2.7.4.1
3.6.1.1
NAD+
H+
1/2O2
2H+
H2O
PPPi
PPi
Pi ADP
3H+
ATP H2O
Ndufa13
Ndufs6
Atp5g1
Atp5b
Atp5g3
Atp5h
Atp5a1
Atp5e
Atp5j2
NADH dehydrogenase
E ND1 ND2 ND3 ND4 ND4L ND5 ND6
E Ndufs1 Ndufs2 Ndufs3 Ndufs4 Ndufs5 Ndufs6 Ndufs7 Ndufs8 Ndufv1 Ndufv2 Ndufv3
B/A NuoA NuoB NuoC NuoD NuoE NuoF NuoG NuoH NuoI NuoJ NuoK NuoL NuoM NuoN
B/A NdhC NdhK NdhJ NdhH NdhA NdhI NdhG NdhE NdhF NdhD NdhB NdhL NdhM NdhN HoxE HoxF HoxU
E Ndufa1 Ndufa2 Ndufa3 Ndufa4 Ndufa5 Ndufa6 Ndufa7 Ndufa8 Ndufa9 Ndufa10 Ndufab1 Ndufa11 Ndufa12 Ndufa13
E Ndufb1 Ndufb2 Ndufb3 Ndufb4 Ndufb5 Ndufb6 Ndufb7 Ndufb8 Ndufb9 Ndufb10 Ndufb11 Ndufc1 Ndufc2
Succinate dehydrogenase / Fumarate reductase
E SDHC SDHD SDHA SDHB
B/A SdhC SdhD SdhA SdhB
FrdA FrdB FrdC FrdD
Cytochrome c reductase
E/B/A ISP Cyt b Cyt 1
E COR1 QCR2 QCR6 QCR7 QCR8 QCR9 QCR10
Cytochrome c oxidase
E COX10 COX3 COX1 COX2 COX4 COX5A COX5B COX6A COX6B COX6C COX7A COX7B COX7C COX8
E/B/A COX11 COX15
COX17
B/A CyoE CyoD CyoC CyoB CyoA
CoxD CoxC CoxA CoxB
QoxD QoxC QoxB QoxA
Cytochrome c oxidase , cbb3-type
B I II IV III
Cytochrome bd complex
B/A CydA CydB
F-type ATPase (Bacteria)
beta alpha gamma delta epsilon c a b
F-type ATPase (Eukaryotes)
beta alpha gamma OSCP delta epsilon c a
b e f6 f 8
d f h j k g
V-type ATPase (Prokaryotes)
A B C D E F I K
V-type ATPase (Eukaryotes)
A B C D E F G H
I AC39 54kD S1 lipid
00190 6/1/12
(c) Kanehisa Laboratories

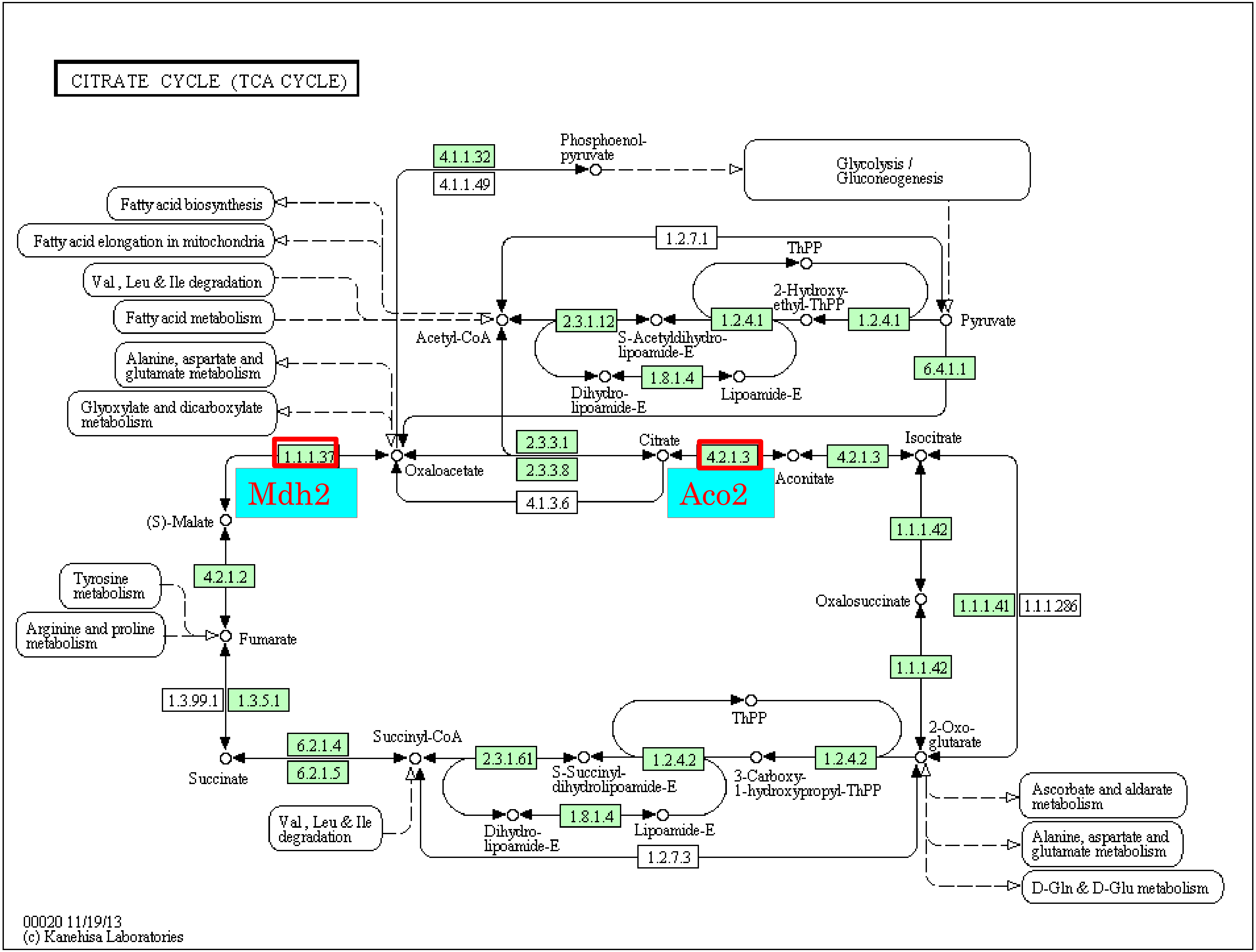

CITRATE CYCLE (TCA CYCLE)
4.1.1.32
4.1.1.49
Phosphoenol-pyruvate
Glycolysis / Gluconeogenesis
Fatty acid biosynthesis
Fatty acid elongation in mitochondria
Val, Leu & Ile degradation
Fatty acid metabolism
Alanine, aspartate and glutamate metabolism
Glyoxylate and dicarboxylate metabolism
1.2.7.1
ThPP
2-Hydroxy-ethyl-ThPP
Acetyl-CoA
2.3.1.12
1.2.4.1
1.2.4.1
Pyruvate
S-Acetyldihydro-lipoamide-E
1.8.1.4
Dihydro-lipoamide-E
Lipoamide-E
6.4.1.1
1.1.1.37
Mdh2
Oxaloacetate
2.3.3.1
2.3.3.8
4.1.3.6
Citrate
4.2.1.3
Aco2
Aconitate
4.2.1.3
Isocitrate
(S)-Malate
4.2.1.2
1.1.1.42
Oxalosuccinate
1.1.1.41
1.1.1.286
1.1.1.42
Tyrosine metabolism
Arginine and proline metabolism
Fumarate
1.3.99.1
1.3.5.1
Succinate
6.2.1.4
6.2.1.5
Succinyl-CoA
2.3.1.61
S-Succinyl-dihydrolipoamide-E
1.2.4.2
1.2.4.2
ThPP
3-Carboxy-1-hydroxypropyl-ThPP
2-Oxo-glutarate
1.8.1.4
Dihydro-lipoamide-E
Lipoamide-E
1.2.7.3
Val, Leu & Ile degradation
Ascorbate and aldarate metabolism
Alanine, aspartate and glutamate metabolism
D-Gln & D-Glu metabolism
00020 11/19/13
(c) Kanehisa Laboratories

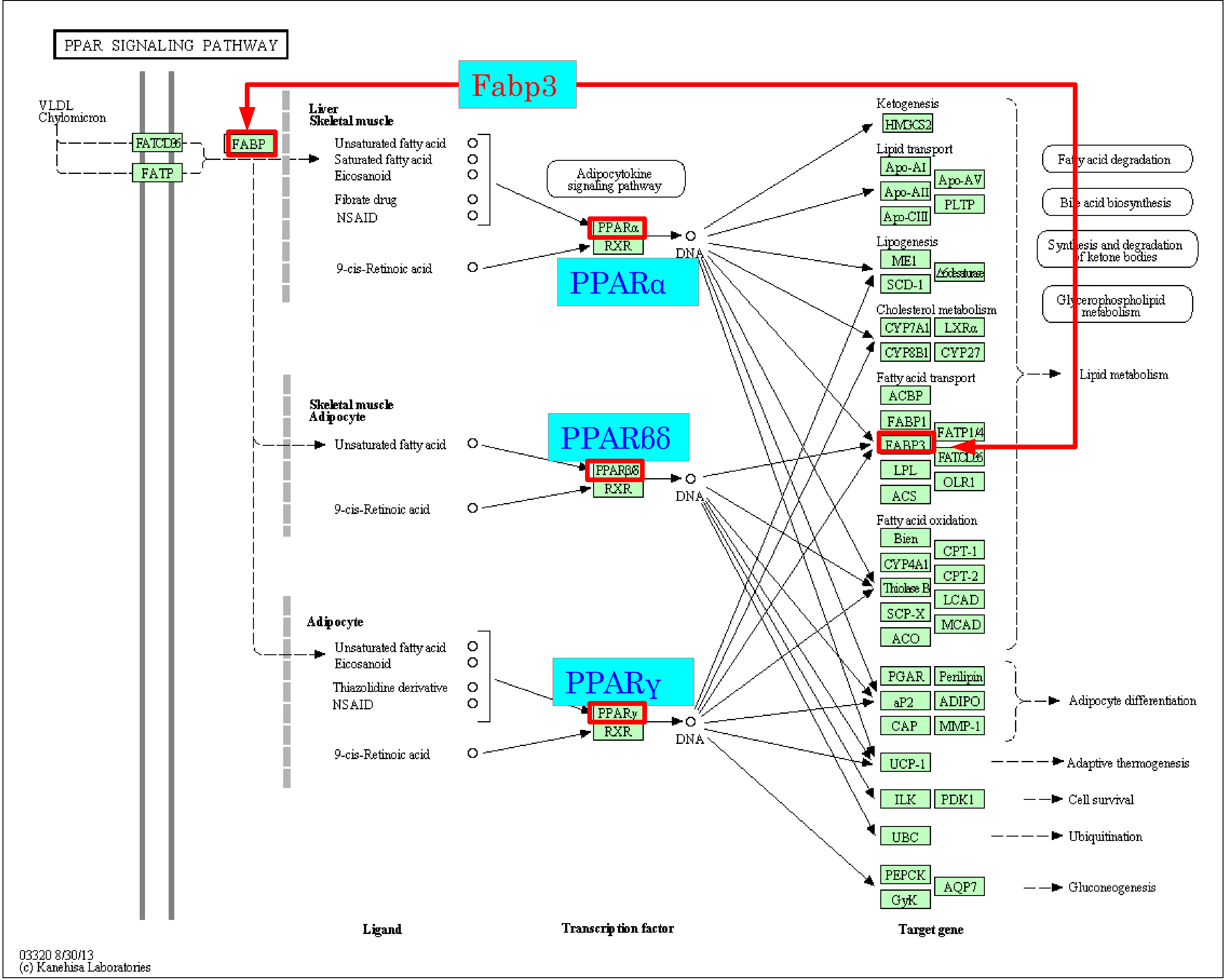
PPAR SIGNALING PATHWAY
Fabp3
VLDL
Chylomicron
FAT/CD36
FATP
FABP
Liver
Skeletal muscle
Unsaturated fatty acid
Saturated fatty acid
Eicosanoid
Fibrate drug
NSAID
9-cis-Retinoic acid
Adipocytokine signaling pathway
PPARα
RXR
DNA
Skeletal muscle
Adipocyte
PPARβδ
Adipocyte
Thiazolidine derivative
PPARγ
Ketogenesis
HMGCS2
Lipid transport
Apo-AI
Apo-AII
Apo-CIII
Apo-AV
PLTP
Lipogenesis
ME1
SCD-1
Δ6desaturase
Cholesterol metabolism
CYP7A1
CYP8B1
LXRα
CYP27
Fatty acid transport
ACBP
FABP1
FABP3
LPL
ACS
FATP1/4
FAT/CD36
OLR1
Fatty acid oxidation
Bien
CYP4A1
Thiolase B
SCP-X
ACO
CPT-1
CPT-2
LCAD
MCAD
PGAR
aP2
CAP
Perilipin
ADIPO
MMP-1
UCP-1
ILK
PDK1
UBC
PEPCK
GyK
AQP7
Fatty acid degradation
Bile acid biosynthesis
Synthesis and degradation of ketone bodies
Glycerophospholipid metabolism
Lipid metabolism
Adipocyte differentiation
Adaptive thermogenesis
Cell survival
Ubiquitination
Gluconeogenesis
Ligand
Transcription factor
Target gene
03320 8/30/13
(c) Kanehisa Laboratories
