# Supplementary material for: Principal component analysis-based unsupervised feature extraction applied to in silico drug discovery for posttraumatic stress disorder-mediated heart disease
Source: BMC Bioinformatics. 2015 Apr 30;16:139. doi: 10.1186/s12859-015-0574-4 (PMC4448281; doi:10.1186/s12859-015-0574-4)
Supplement: Additional file 9 — Stability analysis. Stability analysis of CPCAFE, categorical regression-based FE, and BAHSIC. Frequency represents the number of times each probe was selected among 100 independent ensembles. Number of probes represents the number of probes selected by the corresponding frequency. Numbers in bold represent the number of mRNAs/miRNAs selected 100%. Note that no mRNA was selected 100% by BAHSIC. [file 12859_2015_574_MOESM9_ESM.pdf]

Stability analysis of CPCA-based unsupervised FE											
mRNA											
frequency	1	8	32	36	41	43	73	89	98	99	100
number of probes	1	1	1	1	1	1	1	1	1	1	**78**
miRNA											
frequency	100										
number of probes	**27**										

Stability analysis of categorical regression based FE																				
mRNA																				
frequency	1	2	3	4	5	6	7	8	9	10	11	12	13	14	15	16	17	18	19	20
number of probes	122	61	25	17	15	18	8	9	10	6	6	13	7	6	7	4	4	2	5	1
frequency	21	22	23	24	25	26	27	28	29	30	31	32	33	34	36	37	39	40	42	43
number of probes	7	1	6	5	2	4	6	3	5	5	2	2	3	3	5	3	4	2	4	1
frequency	44	45	46	47	48	49	50	51	55	57	58	60	62	63	65	67	68	71	72	74
number of probes	1	1	4	2	1	3	2	2	1	1	1	1	1	1	1	1	1	1	1	1
frequency	75	77	78	79	82	83	84	87	88	90	94	95	96	97	99	100				
number of probes	1	1	1	3	1	4	1	1	2	1	2	1	1	2	3	**24**				
miRNA																				
frequency	1	2	3	4	5	6	7	8	9	10	11	12	13	14	15	16	17	18	19	20
number of probes	33	29	15	17	3	10	5	8	9	7	2	1	2	4	1	5	2	2	6	1
frequency	21	22	23	24	25	26	27	28	29	30	31	32	33	35	36	37	38	39	40	41
number of probes	2	4	1	5	3	2	4	2	2	4	3	1	1	3	1	1	1	4	3	5
frequency	42	43	44	46	47	48	49	50	52	53	55	56	57	59	60	61	66	67	68	70
number of probes	2	1	2	2	3	1	2	2	2	1	2	3	2	1	4	1	2	2	2	1
frequency	71	74	76	77	78	80	81	82	83	84	86	87	88	89	91	93	95	100		
number of probes	1	2	1	1	1	1	1	1	2	2	1	5	1	2	3	2	2	**8**		

Stability analysis of BAHSIC																
mRNA																
frequency	1	2	3	4	5	6	7	8	9	10	11	12	13	14		
number of probes	2133	886	474	280	197	136	84	55	41	14	7	6	4	1		
miRNA																
frequency	1	3	4	20	21	25	32	40	41	43	45	60	61	62	69	70
number of probes	2	1	1	1	3	1	1	1	1	1	1	1	1	1	1	1
frequency	73	74	75	77	79	82	86	87	90	93	95	96	97	98	99	100
number of probes	3	2	1	3	6	1	1	1	1	1	1	1	3	2	3	**68**
